# Muscle mitochondrial remodeling by intermittent glucocorticoid drugs requires an intact circadian clock and muscle PGC1α

**DOI:** 10.1126/sciadv.abm1189

**Published:** 2022-02-18

**Authors:** Mattia Quattrocelli, Michelle Wintzinger, Karen Miz, Daniel C. Levine, Clara Bien Peek, Joseph Bass, Elizabeth M. McNally

**Affiliations:** 1 Division of Molecular Cardiovascular Biology, Heart Institute, Cincinnati Children’s Hospital Medical Center, Cincinnati, OH, USA.; 2 Department of Pediatrics, University of Cincinnati College of Medicine, Cincinnati, OH, USA.; 3 Center for Genetic Medicine, Feinberg School of Medicine, Northwestern University, Chicago, IL, USA.; 4 Division of Endocrinology, Metabolism and Molecular Medicine, Feinberg School of Medicine, Northwestern University, Chicago, IL, USA.

## Abstract

Exogenous glucocorticoids interact with the circadian clock, but little attention is paid to the timing of intake. We recently found that intermittent once-weekly prednisone improved nutrient oxidation in dystrophic muscle. Here, we investigated whether dosage time affected prednisone effects on muscle bioenergetics. In mice treated with once-weekly prednisone, drug dosing in the light-phase promoted nicotinamide adenine dinucleotide (NAD^+^) levels and mitochondrial function in wild-type muscle, while this response was lost with dark-phase dosing. These effects depended on a normal circadian clock since they were disrupted in muscle from [*Brain and muscle Arnt-like* *protein-1* (*Bmal1*)]–knockout mice. The light-phase prednisone pulse promoted BMAL1-dependent glucocorticoid receptor recruitment on noncanonical targets, including *Nampt* and *Ppargc1a* [peroxisome proliferator-activated receptor-γ coactivator 1α (PGC1α)]. In mice with muscle-restricted inducible PGC1α ablation, bioenergetic stimulation by light-phase prednisone required PGC1α. These results demonstrate that glucocorticoid “chronopharmacology” for muscle bioenergetics requires an intact clock and muscle PGC1α activity.

## Teaser

Light-phase prednisone pulses improve muscle bioenergetics through a BMAL1-dependent GR program to activate muscle PGC1α.

## INTRODUCTION

Glucocorticoids are steroid hormones naturally secreted with a prominent circadian rhythm, peaking at the onset of the active phase, i.e., dark phase in rodents and early light phase in humans (*1*). Exogenous glucocorticoids, e.g., prednisone, are widely used anti-inflammatory drugs, and chronic glucocorticoid intake has a prevalence of >2.5 million people in the United States (*2*). We previously showed that frequency of prednisone dosing, i.e., intermittent once weekly versus the more commonly used once daily, elicited markedly distinct effects in mice with muscular dystrophy (*3*). Unlike daily regimens, weekly intermittent regimens boosted nutrient utilization and mitochondrial bioenergetics in dystrophic muscle (*4*). However, the muscle-autonomous metabolic and circadian determinants of glucocorticoid pharmacology remain unresolved in normal and diseased muscle.

The cognate receptor for these drugs, the glucocorticoid receptor (GR; encoded by the *Nr3c1* gene), is a ligand-activated nuclear receptor that regulates pleiotropic, context-specific cistromes through binding of glucocorticoid-responsive elements (GREs) in gene promoters (*5*). GR regulates and is in turn regulated by core components of the circadian clock (*6*, *7*). At the molecular level, the circadian clock consists of a 24-hour permutation in which the activators, e.g., BMAL1 (diurnally active in mice), induce their own repressors, e.g., Period circadian regulator 2 (PER2) (nocturnally active in mice) (*8*). BMAL1 is an essential component of the diurnal clock complex in mice (*9*) and drives the nicotinamide adenine dinucleotide (NAD^+^)–oxidative capacity in a self-sustained cycle independent from nutrients or feeding (*10*). Other nuclear receptors such as Reverse strand of ERBA (REV-ERBα), encoded by the *Nr1d1* gene, further regulate this time-keeping loop (*11*). In mice, endogenously activated GR (night time) activates transcription of nocturnal factors such as *Per2* (*12*). In turn, the nocturnal cryptochrome factors repress GR activity (*13*). This creates a critical temporal window at the start of the light phase in mice where the GR is highly susceptible to activation by exogenous steroids, likely due to the combination of endogenous corticosterone trough and lack of repressive feedback (*13*, *14*). However, the light phase–specific effects of GR pharmacology remain largely unexplored, particularly in muscle.

Recently, a few studies have shown differential regulation of GR pharmacology in the liver with regard to circadian time of GR activation. The liver is more sensitive to glucocorticoid-induced activation of GR metabolic cistromes during the light than the dark period (*15*). Analyses of liver-specific GR epigenomic landscapes throughout the light and dark periods showed that the GR controls glucose versus triglyceride metabolism through phase-partitioned cistromes in the liver (*16*). However, impact and determinants of time of intake are still unknown for glucocorticoid effects on muscle metabolism. This is remarkable considering the fundamental importance of the circadian clock in regulating mitochondrial function and nutrient utilization in such an energy-demanding tissue (*17*–*20*).

Here, we compared the effects of light-phase versus dark-phase dosage on prednisone effects on muscle bioenergetics. We found that prednisone (single or intermittent pulses) increased NAD^+^ and mitochondrial capacity in muscle when injected at the beginning of the light phase, while these effects were blunted with dark-phase injections. These effects were dependent on BMAL1. Chromatin immunoprecipitation sequencing (ChIP-seq) in muscle after a light-phase prednisone pulse showed convergence of GR and BMAL1 on *Nampt* (NAD^+^ biogenesis) and *Ppargc1a* [peroxisome proliferator-activated receptor-γ coactivator 1α (PGC1α); mitochondrial biogenesis] promoters. The light-phase prednisone effects on muscle NAD^+^ were blunted by nicotinamide phosphoribosyltransferase (NAMPT) inhibition, and the effects on mitochondrial capacity were blunted by muscle-specific inducible PGC1α knockout (KO). Moreover, immunoprecipitation (IP) and confocal analyses showed that light-phase prednisone promoted GR and BMAL1 cross-interaction and nuclear translocation in muscle. Our study provides evidence for circadian-dependent mechanisms of exogenous glucocorticoid effects on muscle mitochondrial function, identifying epigenetic and circadian mechanisms to account for their role as bioenergetic facilitators of skeletal muscle.

## RESULTS

### The effects of intermittent prednisone on muscle NAD^+^ and bioenergetics depend on dosage time and BMAL1

We previously showed that once-weekly intermittent dosing of glucocorticoids such as prednisone improves mitochondrial bioenergetics in dystrophic muscle (*4*). The GR is differentially susceptible to exogenous steroid activation according to circadian time, with a notable peak of susceptibility at the light-phase start in mice (*13*). We therefore asked whether dosage time determined the bioenergetic effects of chronic intermittent glucocorticoids in muscle. We focused on wild-type (WT) adult muscle to study the regimen effects in the absence of pathology. We used prednisone as exogenous glucocorticoid in line with our previous studies with dystrophic mice because its short half-life makes it amenable to discern time-of-dose effects in vivo (*21*). We treated WT mice (12 weeks of age) with a 12-week-long regimen of 1×/weekly prednisone (1 mg/kg) or vehicle. As mice were maintained on a 14-hour light/10-hour dark cycle, we compared regimens with dosing at the start of the light phase [Zeitgeber time 0 (ZT0), lights on] versus start of the dark phase (ZT14, lights off). Analyses were conducted at 24 hours after last injection.

**Fig. 1.**
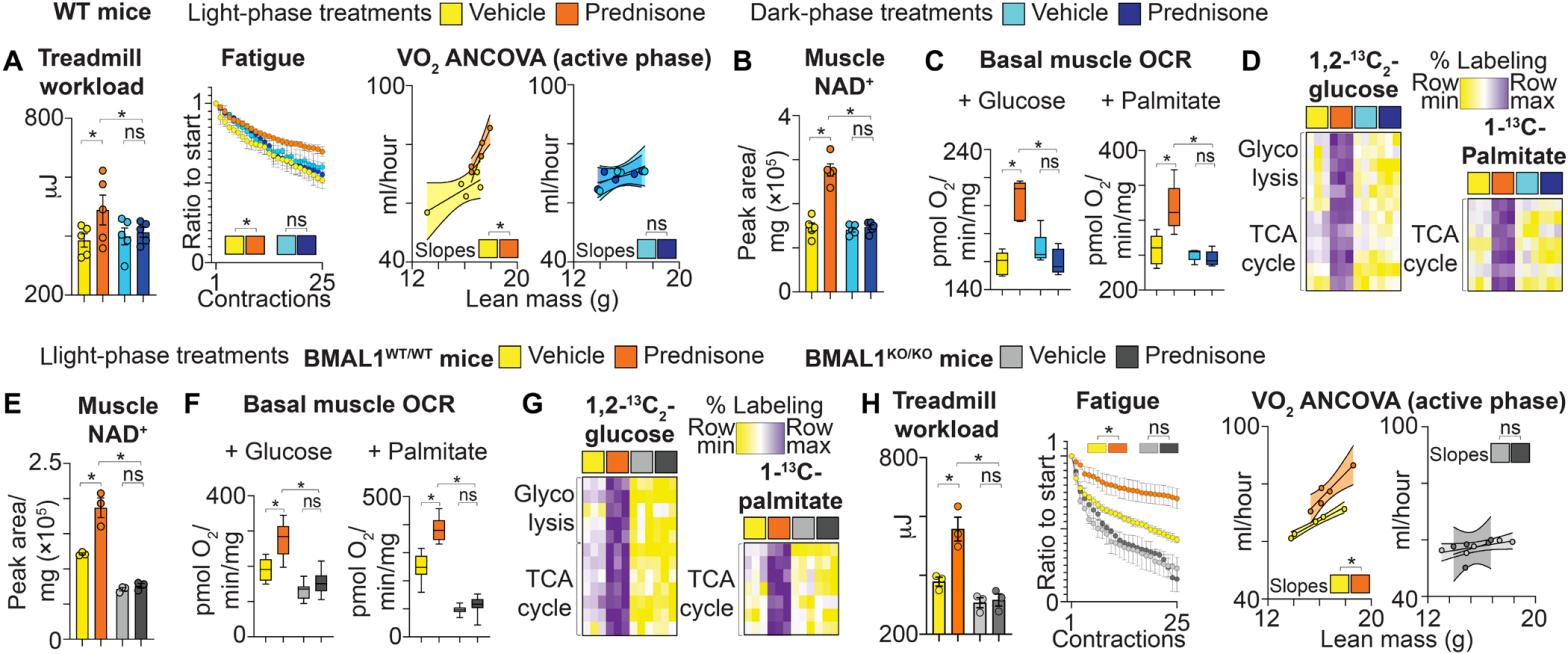
BMAL1 is required for the effects of intermittent light-phase prednisone on muscle bioenergetics. Results are shown after a 12-week-long treatment with intermittent once-weekly prednisone with dosing restricted to ZT0 (light phase) versus ZT14 (dark phase). (**A**) In WT mice, compared to isochronic vehicle controls, ZT0, but not ZT14, prednisone improved treadmill performance, muscle fatigue (in situ tibialis anterior), and body-wide VO_2_ normalized to lean mass. (**B** and **C**) This correlated with gains in muscle NAD^+^ (mass spectrometry) and basal OCR in muscle tissue (Seahorse respirometry) after ZT0, but not ZT14, regimens. (**D**) ZT0, but not ZT14, prednisone increased in muscle catabolism of either glucose or palmitate, as shown by steady-state ^13^C tracing in ex vivo contracting muscle. (**E** to **G**) Compared to BMAL1-WT littermates, BMAL1-KO mice blunted the gain in muscle NAD^+^ induced by ZT0 prednisone. This correlated with analogous trends in basal respiration and ^13^C-labeled nutrient oxidative catabolism in muscle tissue. (**H**) The effects of ZT0 prednisone treatment on treadmill performance, muscle fatigue, and VO_2_ in BMAL1-WT mice were blocked in BMAL1-KO mice. *n* = 5 (male, female) per group for (A) to (D); *n* = 3 (male, female) per group for (E) to (I). *P < 0.05, one-way analysis of variance (ANOVA) + Sidak (histograms) and two-way ANOVA (curves). ns, not significant.

Compared to time-matched vehicle controls, light-phase, but not dark-phase, prednisone exposure increased muscle endurance, seen as treadmill performance, and improved fatiguability to repetitive in situ contractions in tibialis anterior muscles (Fig. 1A). Increased endurance correlated with increased VO_2_ capacity normalized to lean mass (Fig. 1A). These effects correlated with gains in muscle NAD^+^ content and muscle tissue respiration with light-phase, but not dark-phase, treatments, as determined respectively by mass spectrometry (MS) and Seahorse respirometry (Fig. 1, B and C). Dosage time-dependent effects on nutrient oxidation were confirmed through analysis of ^13^C labeling ratio in catabolic intermediates of macronutrients in ex vivo contracting muscle (Fig. 1D). The gain in muscle oxidative capacity was not nutrient-restricted, as light-phase treatment increased respiration and ^13^C labeling with both glucose and palmitate, used as exclusive fuels in their respective assays (Fig. 1, C and D). Thus, dosage at the start of the light period (ZT0) significantly increased the effects of intermittent prednisone regimens on muscle bioenergetics and performance.

Considering the effects on muscle performance, we quantified treatment effects on muscle mass, myofiber typing, and force. Light-phase prednisone increased lean mass, muscle mass, and myofiber cross-sectional area, correlating with increased grip strength and muscle force (fig. S1, A to D). These effects were blocked by dark-phase injections, and treatments had no overall effects on body weight (fig. S1A). No changes were noted in contraction/relaxation times during tetanic contraction or in the distribution of myofiber types in tibialis anterior muscles (fig. S1, C and D). Furthermore, we asked whether the endogenous circadian rhythm of mice was altered by the intermittent treatments. Plasma levels of endogenous corticosterone were unchanged by treatments at 24 hours after last injection, as measured in rest phase (trough) and active phase (peak) and compared to vehicle-treated mice (fig. S1E). We assessed the circadian fluctuations in activity and food intake in metabolic cages. Albeit increased in amplitude, the VO_2_/lean mass curve did not show treatment-dependent changes in peak timing. Analogously, we did not find significant changes in circadian fluctuations of spontaneous locomotion in the cage and food intake among cohorts (fig. S1F). Together, these data indicate that light-phase intermittent prednisone promoted muscle function without apparent dysregulation of circadian rhythm.

We were intrigued by the correlation between light-phase dosing and elevation of NAD^+^ and mitochondrial capacity in muscle. In peripheral tissues in mice, the diurnal (resting) phase is clocked by BMAL1 (encoded by *Arntl*) activity, an essential component of the circadian clock core (*9*). BMAL1 promotes NAD^+^ and mitochondrial capacity (*10*). We therefore asked whether BMAL1 was required for the metabolic effects of intermittent prednisone in muscle. We treated *Arntl^−/−^* (BMAL1-KO) versus *Arntl^+^/^+^* (BMAL1-WT) littermates on the BL6 background for 12 weeks with light phase–restricted (injections at ZT0) intermittent prednisone from the age of 12 weeks. This allowed us to complete the study before onset of prominent wasting in these mice after 6 months of age (*22*). Unlike BMAL1-WT control, BMAL1-KO muscle failed to up-regulate muscle NAD^+^ after prednisone treatment, as compared to vehicle (Fig. 1E). Compared to vehicle, light-phase prednisone treatment increased basal respiration and ^13^C-labeled nutrient oxidative catabolism in muscle tissue in BMAL1-WT, but not in BMAL1-KO, mice (Fig. 1, F and G). Consistent with the trends in mitochondrial function, the effects of light-phase prednisone treatment on treadmill performance, muscle fatigue, and VO_2_ in BMAL1-WT mice were blocked in BMAL1-KO mice (Fig. 1H). Absence of BMAL1 also blocked the treatment-induced effects on muscle mass and force (fig. S1, G and H). Furthermore, untargeted hydrophilic metabolomics of muscle tissue (not subjected to ex vivo contractions and ^13^C nutrient exposure) showed a treatment-dependent gain of glycolytic intermediates, tricarboxylic acid (TCA) cycle metabolites, adenosine 5′-triphosphate (ATP), and phosphocreatine in BMAL1-WT, but not BMAL1-KO, muscle (fig. S1I). Thus, BMAL1 was required for light-phase intermittent prednisone effects on muscle NAD^+^ and bioenergetics.

### Light-phase prednisone pulse increases BMAL1-dependent GR recruitment to *Nampt* and *Ppargc1a* promoters in muscle

**Fig. 2.**
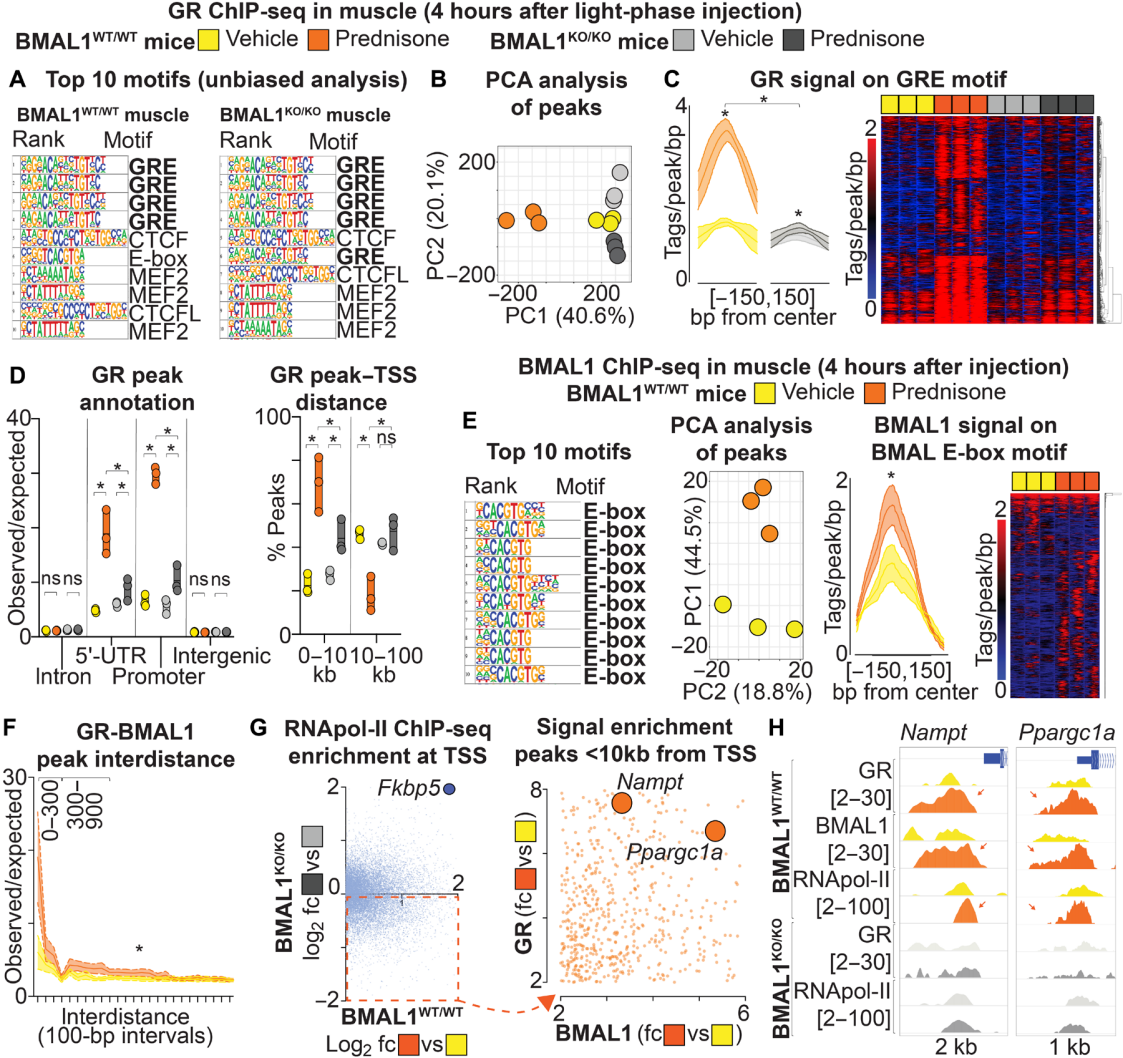
A light-phase prednisone pulse increases BMAL1-dependent GR recruitment to *Nampt* and *Ppargc1a* promoters in muscle. Results are shown at 4 hours (ZT4) after a single prednisone pulse in vivo at ZT0. (**A**) Unbiased motif analysis validated the muscle ChIP-seq for GR in both BMAL1-WT and BMAL1-KO quadriceps muscles. (**B**) Muscle GR peak profiles clustered according to genotype and drug. (**C**) Genome-wide GR occupancy of GRE sites was increased by the drug pulse. This effect was strongly limited in the BMAL1-KO muscle. (**D**) Prednisone shifted the muscle GR peaks from distal (>10 kb) to proximal (<10 kb) regions from TSSs, correlating with an enrichment in GR peaks in 5′UTR and promoter regions. These trends were partially blunted in BMAL1-KO muscle. (**E**) BMAL1 ChIP-seq in muscle showed enrichment for E-box motif in signal peaks. Muscle BMAL1 occupancy of E-box sites increased after prednisone pulse. (**F**) In BMAL1-WT muscle, the drug pulse increased the cooccurrence of peaks of GR and BMAL1 in the 0– to 300–base pair (bp) and 300- to 900-bp ranges. (**G** and **H**) Among genes with a BMAL1-dependent gain of RNA polymerase II (RNApol-II) at TSS with drug pulse, *Nampt* and *Ppargc1a* showed enrichment for both GR and BMAL1 signal in the promoter. The drug pulse increased GR, BMAL1, and RNApol-II peaks (arrows) on *Nampt* and *Ppargc1a* promoters in BMAL1-WT muscle, but not in BMAL1-KO muscle. *N* = 3 ♂ per group. *P < 0.05, two-way ANOVA + Sidak. fc, fold change.

The transcription factors GR and BMAL1 are expressed in muscle (*23*, *24*). To assess the epigenomic effects of prednisone, we injected BMAL1-WT and BMAL1-KO mice with a single pulse of prednisone [1 mg/kg, intraperitoneally (i.p.)] or vehicle at ZT0 and then harvested the muscles after 4 hours (ZT4). This was the same timing used in prior work on light-phase GR activation with exogenous steroids in the liver (*16*). We performed GR ChIP-seq from whole quadriceps muscle tissue chromatin. Unbiased analysis of GR peaks in both BMAL1-WT and BMAL1-KO muscles showed enrichment for the GRE motif in top ranked motifs, validating our datasets (Fig. 2A). Principal components analysis (PCA) analysis of GR peaks showed sample clustering according to genotype and treatment (Fig. 2B). As expected, compared to vehicle, light-phase prednisone induced GR activity, as quantitated by the gain in relative GR signal on GRE sites genome-wide. Notably, the drug-driven GR epigenomic activity was significantly reduced in BMAL1-KO muscle (Fig. 2C). Annotating GR peaks for genomic region and for distance to transcription start site (TSS) in BMAL1-WT muscle, we found that light-phase prednisone increased the GR peaks mainly in 5′ untranslated region (5′UTR) and promoter regions, shifting the enrichment from >10- to <10-kb distance from TSSs. These trends were significantly reduced in BMAL1-KO muscle, indicating that BMAL1 regulated the GR epigenomic activity in muscle in response to the light-phase prednisone pulse (Fig. 2D). In addition, we found that BMAL1 deletion affected the GR activity even with respect to peak number, which was significantly reduced in BMAL1-KO versus BMAL1-WT muscles both after vehicle and drug pulses (fig. S2A). In BMAL1-WT, but not in BMAL1-KO, muscle, we found that the E-box motif (target motif for BMAL1 binding to the DNA) was present in the GR-bound regions (fig. S2B), suggesting the possibility of overlapping or close-range occupancy between GR and BMAL1 on muscle chromatin.

We then performed BMAL1 ChIP-seq on the same muscle chromatin samples from BMAL1-WT muscles after vehicle versus light-phase prednisone pulses. Unbiased motif analysis showed a strong enrichment in E-box motif in BMAL1 peaks validating our datasets, while PCA analysis clustered the samples according to treatment (Fig. 2E). Notably, compared to vehicle, prednisone increased muscle BMAL1 activity, as quantitated by the gain in relative BMAL1 signal on E-box sites genome wide (Fig. 2E). The drug pulse also increased BMAL1 peaks in promoter regions, shifting the enrichment from >10- to <10-kb distance from TSSs (fig. S2C). We cross-analyzed the GR and BMAL1 ChIP-seq datasets for evidence of convergent activity between the two transcription factors after drug pulse. Quantitating the peak interdistance, we found that the drug pulse increased the occurrence of GR and BMAL1 peaks within the 0– to 300–base pair (bp) and 300- to 900-bp intervals, suggesting increased binding in overlapping or close-range regions (Fig. 2F). Accordingly, we quantitated the relative GR signal on E-box (BMAL1-bound motif) and the relative BMAL1 signal on GRE (GR-bound motif). Both were increased by the drug pulse compared to vehicle (fig. S2D). To investigate whether the convergence of GR and BMAL1 was linked to immediate changes in transcriptional activity, we performed RNA polymerase II (RNApol-II) ChIP-seq on the same muscle chromatin samples from BMAL1-WT versus BMAL1-KO muscles after vehicle versus light-phase prednisone pulses. Quantitating RNApol-II signal on TSSs, we screened genes for RNApol-II gain of signal (transcriptional up-regulation) induced by the drug pulse in either BMAL1-WT muscle or BMAL1-KO muscle (drug versus vehicle comparison per genotype). As control, we found that the canonical marker of glucocorticoid-GR axis activation *Fkbp5* showed drug-driven gain of RNApol-II signal in both BMAL1-WT and BMAL1-KO muscles (Fig. 2G). We then selected 5961 genes that showed a BMAL1-dependent, drug-driven up-regulation (up-regulated with prednisone versus vehicle in BMAL1-WT, but not in BMAL1-KO, muscles; Fig. 2G, dotted quadrant). We screened these genes for drug-driven gain of GR and BMAL1 signal within 10 kb from their TSS (table S1), considering that both GR and BMAL1 peaks were enriched at <10 kb from TSSs by the drug pulse (see above). Among the genes with peak signal gain for both factors, gene ontology analysis revealed enrichment in circadian, metabolic, and muscle function pathways (fig. S2E). Among the genes shared by the enriched gene ontology pathways, we found *Nampt* and *Ppargc1a* as marked by GR-BMAL1 signal enrichment (Fig. 2G, right). We focused on these two genes due to the light-phase prednisone effects on NAD^+^ and mitochondrial capacity in muscle. *Nampt* encodes the NAD^+^-producing enzyme NAMPT (*25*), and *Ppargc1a* encodes the mitochondrial regulator PGC1α (*26*). Peak tracks showed that light-phase prednisone increased GR, BMAL1, and RNApol-II signal in the proximal promoter regions upstream of both *Nampt* and *Ppargc1a* in BMAL1-WT muscle, while drug-driven gains in GR and RNApol-II signal were lost in BMAL1-KO muscle (Fig. 2H). Unlike *Nampt* and *Ppargc1a*, we found that the BMAL1 canonical targets in the clock core machinery showed variable responses to the drug pulse. *Nr1d1* (encoding REV-ERBα and BMAL1 repressor) did not show drug-driven changes in peak signal (fig. S2F). The promoter of *Per2*, canonical target of both GR and BMAL1, showed a ~2-fold increase in GR occupancy after drug pulse, but no changes in BMAL1 binding. BMAL1-KO did not change GR enrichment but reduced RNApol-II recruitment to its TSS (fig. S2F). We also did not find changes in total GR protein levels between vehicle- and prednisone-pulsed BMAL1-WT and BMAL1-KO muscles (fig. S2G), confirming that the GR epigenomic regulation was dependent on its activation rather than overall level changes. Thus, in aggregate, these data show that light-phase prednisone promoted epigenomic convergence of GR and BMAL1 in muscle. Moreover, BMAL1 was required for drug-induced gain of GR promoter occupancy and transcriptional up-regulation of *Nampt* and *Ppargc1a* in muscle.

To verify that the up-regulation of *Nampt* and *Ppargc1a* was dependent on dosage in the light period, we monitored gene expression levels in muscle in vivo after a light-phase (ZT0) versus dark-phase (ZT14) intraperitoneal prednisone pulse. We analyzed expression levels in quadriceps muscle through quantitative polymerase chain reaction (qPCR) every 4 hours over a circadian period, starting at 1 hour after injection. Light-phase, but not dark-phase, prednisone increased *Nampt* and *Ppargc1a* expression over their normal fluctuations. No significant changes were found in *Nr1d1* expression, while the up-regulation of *Per2* by the drug pulse was briefer with light-phase than with dark-phase prednisone (fig. S2H). Moreover, as BMAL1 activity peaks at ZT8 in mice, we compared levels of *Nampt* and NAD^+^ induction in muscle by prednisone injections at ZT0, ZT4, and ZT8 at 24 hours after injection. We found nonsignificant trends in enhanced induction with the dosing time nearing ZT8 (fig. S2I). Furthermore, we performed ChIP-qPCR for GR and BMAL1 occupancy on the promoter regions of *Nampt* and *Ppargc1a* that were enriched for GR and BMAL1 peaks (Fig. 2H) in muscle at end point of 12-week-long light-phase intermittent prednisone. At 4 hours after last injection, GR and BMAL1 occupancy of *Nampt* and *Ppargc1a* promoters was increased by light-phase intermittent prednisone in BMAL1-WT muscle. BMAL1-KO mice showed reduced GR occupancy in those regulatory regions in vehicle-treated muscle and no treatment-induced gains (fig. S2J). Thus, the BMAL1-dependent epigenetic effects of a single pulse of light-phase prednisone correlated with transient *Nampt* and *Ppargc1a* up-regulation and were maintained in the chronic intermittent treatment.

### Muscle PGC1α mediates the mitochondrial effects of a light-phase prednisone pulse in vivo

**Fig. 3.**
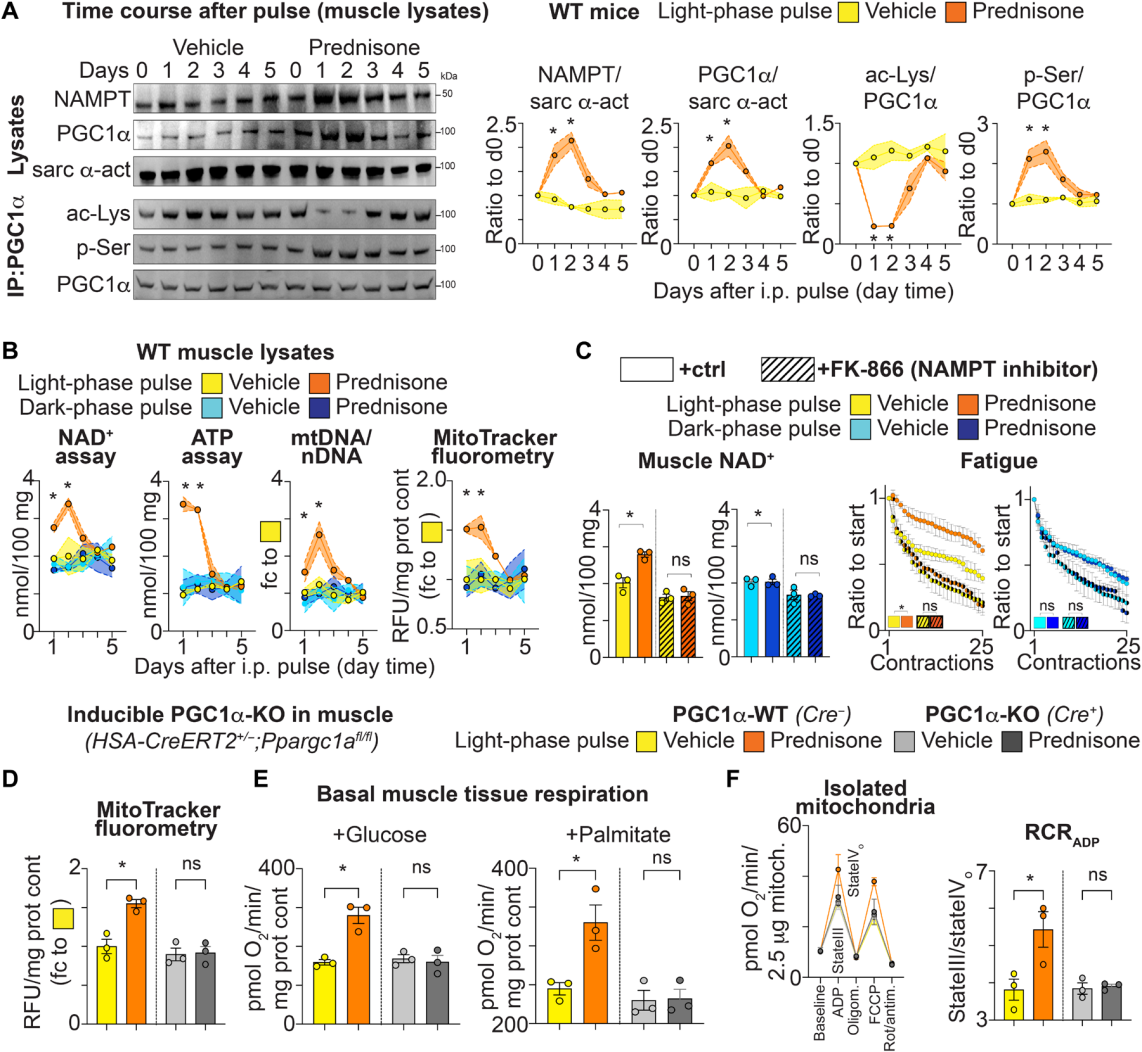
A light-phase pulse of prednisone transiently increases NAD^+^ and mitochondrial capacity in muscle through NAMPT activity and PGC1α. Results are shown after a single prednisone pulse in vivo at ZT0 (light phase) or ZT14 (dark phase). (**A**) Compared to vehicle, the ZT0 prednisone pulse increased total levels of NAMPT and PGC1α, as well as PGC1α deacetylation and phosphorylation, at days 1 and 2 after injection. (**B**) Analogous trends were observed for muscle NAD^+^, ATP, and mitochondrial density. The latter was quantitated through both mtDNA/nDNA ratio in muscle tissue and normalized MitoTracker fluorometry in isolated myofibers. The gains at days 1 and 2 after pulse were lost with ZT14 dosing. RFU, relative fluorescent units. (**C**) Coinjection with the NAMPT inhibitor FK-866 blunted the ZT0 prednisone effects on NAD^+^ and muscle fatigue. (**D** to **F**) After induction of genetic PGC1α ablation in adult muscle, the gain in muscle mitochondrial density induced by the ZT0 prednisone pulse was blocked. This correlated with analogous trends on basal respiration and mitochondrial RCR (stateIII/stateIV_o_) of muscle. FCCP, carbonyl cyanide *p*-trifluoromethoxyphenylhydrazone. *N* = 3 (♂, ♀) per group per time point. *P < 0.05, two-way ANOVA + Sidak for curves and one-way ANOVA + Sidak for histograms.

We next sought to determine whether the epigenomic effects of a light-phase prednisone pulse translated in protein and bioenergetic changes in muscle in vivo. NAMPT generates NAD^+^, which then serves as substrate for sirtuin-mediated deacetylation of PGC1α. This primes PGC1α to be phosphorylated and activated (*27*). We treated WT mice (12 weeks of age) with a single intraperitoneal injection of prednisone (1 mg/kg) versus vehicle at ZT0 and followed NAMPT and PGC1α protein levels in quadriceps muscle at 1-2-3-4-5 days after injection (analyses at ZT0 for each time point). Compared to vehicle, light-phase prednisone increased total levels of NAMPT and PGC1α for 2 days after pulse. Moreover, from the same lysates, we immunoprecipitated PGC1α and immunoblotted for acetyl-lysine (ac-Lys) and phospho-serine (p-Ser) levels. For 2 days after injection, the drug pulse reduced PGC1α acetylation while increasing its phosphorylation (Fig. 3A). In addition, gain in total PGC1α at 24 hours after injection was mirrored by similar gain in nuclear PGC1α levels in muscle, further supporting increased PGC1α activation (fig. S3A). Thus, the epigenomic effects on *Nampt* and *Pparcgc1a* promoters translated into a transient spike in NAMPT and PGC1α levels in muscle, with increased activation profile of PGC1α (loss of acetylation/gain of phosphorylation).

We then investigated the extent to which circadian dosage time affected the drug pulse effects on muscle bioenergetics in vivo. We repeated the in vivo pulse experiment comparing light-phase (ZT0) versus dark-phase (ZT14) pulses of prednisone or vehicle. Analyses were carried at the injection-matching circadian time at 1-2-3-4-5 days after injection. Compared to vehicle, light-phase, but not dark-phase, prednisone increased NAD^+^ and ATP in quadriceps muscle for 2 days after injection. Considering the protein and posttranslational changes in PGC1α, we also quantitated mitochondrial density through two parallel assays, mitochondrial DNA/nuclear DNA (mtDNA/nDNA) ratio and unbiased MitoTracker Green FM fluorometry in isolated myofibers. MitoTracker Green FM signal is independent from mitochondrial membrane potential (*28*). Both assays showed gain of mitochondrial density signal for two days after a light-phase, but not a dark-phase, prednisone pulse (Fig. 3B), confirming the dosage time impact on the effects of a glucocorticoid pulse on muscle mitochondrial density.

In addition, we asked whether the observed gain in NAD^+^ was due to NAD^+^ biogenesis. Together with the light-phase prednisone/vehicle pulses, we coinjected WT mice with FK-866, a specific NAMPT antagonist (*29*). At 24 hours after injection, FK-866 blunted the drug-driven effects (drug versus vehicle) on NAD^+^ gain in muscle tissue with light-phase, but not dark-phase, prednisone, correlating with analogous trends in muscle fatigue (Fig. 3C). Similar trends were also found in in situ muscle force production (fig. S3B). The effects of light-phase prednisone on muscle NAD^+^ appeared dependent on biogenesis and correlated with force development.

We were intrigued by the transient up-regulation of total and activated PGC1α following a light-phase prednisone pulse. We asked whether this was required by the time-restricted drug pulse effects on mitochondrial density and respiration. We generated transgenic mice for muscle-restricted inducible ablation of PGC1α by crossing *Ppargc1a^fl/fl^* (*30*) with *HSA-CreERT2^+/−^* mice (*31*) on the C57BL/6 background. At 12 weeks of age, we induced PGC1α ablation through a sequence of intraperitoneal tamoxifen injections (20 mg/kg per day for 5 days), followed by 14 days on tamoxifen-containing chow (40 mg/kg) and 2 days of regular chow for tamoxifen washout. This induction paradigm ablated ~85% of PGC1α in quadriceps muscle but not left ventricle without macroscopic signs of toxicity (fig. S3C). We therefore compared PGC1α-WT (*Ppargc1a^fl/fl^; HSA-CreERT2^−/−^*) versus PGC1α-KO (*Ppargc1a^fl/fl^; HSA-CreERT^+/−^*) littermates for the effects of light-phase prednisone in vivo immediately after tamoxifen exposure, i.e., at 12 weeks of age. We injected PGC1α-WT and PGC1α-KO mice with a single intraperitoneal prednisone dose (1 mg/kg) at ZT0 (light-phase) and monitored the mitochondrial effects at 24 hours after dose. PGC1α ablation blunted the effects of light-phase prednisone (drug versus vehicle) on muscle mitochondrial density, as shown by MitoTracker fluorometry in isolated myofibers (Fig. 3D). Unlike PGC1α-WT muscle, PGC1α-KO muscle failed to up-regulate basal tissue respiration in muscle (Seahorse respirometry) with either glucose or palmitate (Fig. 3E). Analogous trends were found with the respiratory control ratio (RCR) of isolated mitochondria from quadriceps muscle (RCR_ADP_; stateIII/stateIV_o_; Fig. 3F). The RCR measures overall respiratory function, i.e., substrate oxidation for adenosine 5′-diphosphate (ADP) phosphorylation, in isolated mitochondria (*32*, *33*). It must be noted that at baseline, no sizable changes were found in mitochondrial density or respiration between PGC1α-WT and PGC1α-KO muscles [compare vehicle data from both genotypes in Fig. 3 (D to F)]. Conversely, no genotype or drug effects were observed in these mitochondrial parameters when prednisone was pulsed in vivo with a dark-phase (ZT14) dose (fig. S3D). In aggregate, these data show that a light phase–restricted prednisone pulse transiently boosted mitochondrial capacity in muscle through PGC1α.

### Light-phase prednisone pulse promotes GR and BMAL1 cross-interaction and nuclear translocation

**Fig. 4.**
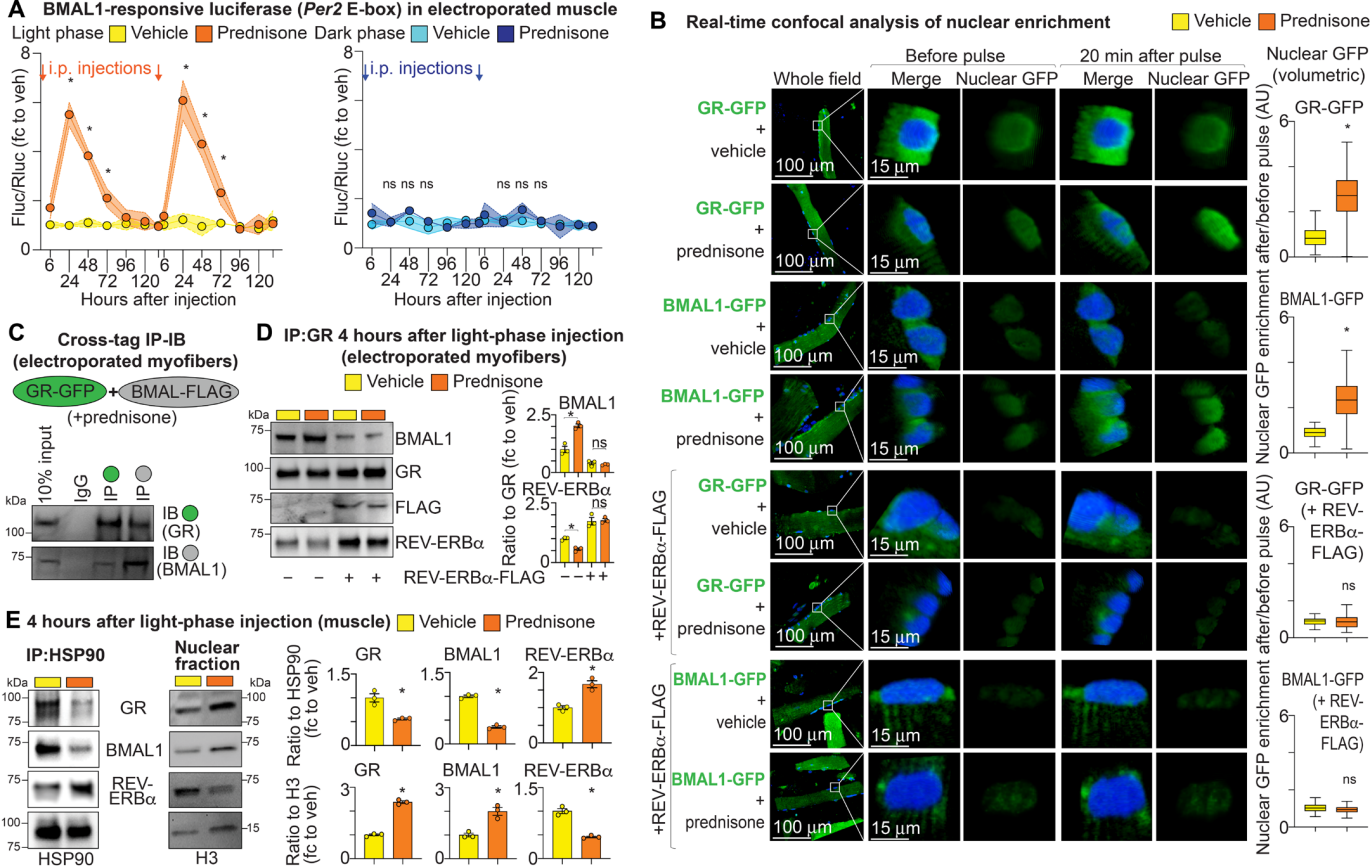
A light-phase pulse of prednisone promotes GR and BMAL1 cross-interaction and nuclear translocation. Results are shown after drug pulses in vivo (A and C to E) and ex vivo (B). (**A**) Mice with muscles electroporated with a BMAL1-responsive luciferase vector in vivo showed a transient increase in luciferase activity after two intermittent pulses of ZT0 prednisone compared to vehicle. The trends were blunted with ZT14 drug pulses. (**B**) Once isolated ex vivo at ZT0, myofibers showed increased translocation of GFP-tagged GR and BMAL1 isoforms at 20 min after prednisone exposure. The trends were blocked by cotransfection with an REV-ERBα–overexpressing construct. AU, arbitrary units. (**C**) Cross-tag IP in myofibers electroporated with the same tagged vectors used for imaging (overexpression system) showed interaction between GR and BMAL1 with prednisone. IB, immunoblotting. (**D**) IP of endogenous GR from muscle tissue showed increased binding of BMAL1 at 4 hours (ZT4) after in vivo pulse at ZT0. The drug-driven effect was blocked by REV-ERBα overexpression. (**E**) At ZT4 (4 hours after pulse), ZT0 prednisone decreased the HSP90-bound cytoplasmic GR and BMAL1 (endogenous) in muscle tissue while promoting their nuclear levels. Endogenous REV-ERBα showed opposite trends. *N* = 3 (♂, ♀) per group per time point. *P < 0.05, two-way ANOVA + Sidak for curves, Welch’s *t* test for (B) and (E), and one-way ANOVA + Sidak for (D).

Glucocorticoids regulate the circadian clock, and the activated GR can directly up-regulate expression of clock factors such as PER2. Our previous experiments point at a cross-talk between the GR and the activating clock forelimb factor BMAL1 in muscle, triggered by a light phase–restricted prednisone pulse. We therefore asked whether time of dosage shifted the drug effects on muscle BMAL1 activity. We monitored in vivo fluctuations of BMAL1 activity through the luciferase vector containing the proximal BMAL1-responsive E-box in *Per2* promoter (*34*). The regulatory region cloned in this reporter construct does not contain the GR-binding sites for *Per2* (*12*). We electroporated the flexor digitorum brevis (FDB) muscles of WT mice and probed luciferase activity in the muscle lysates at 24, 48, 72, 96, and 120 hours after a light-phase (ZT0) versus dark-phase (ZT14) prednisone pulse in vivo. In the absence of prednisone, the luciferase signal in FDB myofibers was higher at ZT8 than ZT20, confirming the BMAL1-reporting activity (fig. S4A). Considering our results with intermittent weekly regimens, we treated a subset of mice with the first pulse and then with a subsequent pulse after a week, monitoring luciferase after each pulse. Compared to vehicle, both first and second intraperitoneal light-phase prednisone pulses increased the relative BMAL1-responsive luciferase activity in muscle with a transient spike at 24 hours after pulse and a downward trail at 48 and 72 hours, while these effects were not seen with dark-phase prednisone pulses (Fig. 4A). We then asked whether this transient spike in BMAL1 activity was changing the amplitude or shifting the period of muscle clock oscillations. We electroporated muscles of WT mice with the same BMAL1-responsive construct, and we treated the mice with a light-phase versus dark-phase prednisone pulse in vivo. We then explanted the electroporated muscles at 2 hours after injection, when glucocorticoids are already bioavailable in muscle (*21*), and we analyzed them at the LumiCycle for continuous scan of luciferase signal from the whole intact muscle. Muscle clock was not reset with further drug exposure ex vivo. This procedure allowed us to interrogate the luciferase reporter activity as close as possible to the in vivo pharmacology effects. In contrast to the dark-phase pulse, the light-phase prednisone pulse transiently increased the amplitude of BMAL1-responsive luciferase oscillations (fig. S4B). The oscillation period was not significantly changed by the drug pulses, suggesting that the intrinsic feedback loops of the peripheral clock were not disrupted despite the transient gain in amplitude (fig. S4B). Thus, the diurnal time of prednisone dosing restricted the drug pulse effects on BMAL1 activity in muscle.

Considering the gain in epigenomic activity for both GR and BMAL1 quantitated in our ChIP-seq analyses, we asked whether light-phase prednisone promoted nuclear translocation of both factors. We quantitated this through green fluorescent protein (GFP)–tagged GR and BMAL1 constructs in electroporated muscles. We isolated myofibers from the electroporated muscles at ZT0 and analyzed the enrichment of nuclear-restricted GFP through real-time confocal microscopy. We imaged the myofibers at 0 and 20 min after a prednisone pulse (25 μg/ml) in vitro. We collected z-stacks and quantitated the nuclear GFP through volumetric GFP signal filtering through Hoechst signal. We then analyzed the post/pre signal ratio, comparing vehicle to prednisone. At 20 min after ex vivo drug exposure, prednisone promoted nuclear translocation of both tagged GR and BMAL1 constructs (Fig. 4B, top half). In hepatocytes, the clock factor REV-ERBα has been shown to counteract GR translocation from cytoplasm to nucleus (*35*). REV-ERBα is part of the clock feedback loop repressing the activating BMAL1/Circadian Locomotor Output Cycles Kaput (CLOCK) complex (*8*). We confirmed the inhibitory activity of REV-ERBα overexpression in muscle on nuclear localization of both exogenous and endogenous GR and BMAL1 in the absence of prednisone (fig. S4C). We then asked whether overexpression of REV-ERBα counteracted the drug effect on translocation of GR and BMAL1 in muscle. In myofibers coelectroporated with the GFP-tagged constructs and a REV-ERBα expression construct, the drug-dependent effects on GR and BMAL1 nuclear translocation were ablated (Fig. 4B, bottom half). Thus, light-phase prednisone promoted nuclear translocation of GR and BMAL1 in muscle, and this was counteracted by REV-ERBα overexpression.

Considering the gain in close-range and overlapping GR and BMAL1 peak signal indicated by the ChIP-seq datasets, we asked whether light-phase prednisone promoted physical GR-BMAL1 interaction in muscle. We first probed this through cross-tag IP in electroporated muscles with the same vectors used for nuclear translocation imaging. In muscle cooverexpressing GR-GFP and BMAL1-FLAG lysed 4 hours after a light-phase prednisone intraperitoneal pulse, we found coprecipitation after either GFP- or FLAG-based pull-down (Fig. 4C). We validated this approach through a cross-tag IP experiment with Krüppel-like factor 15 (KLF15), which is known to interact with the GR (fig. S4D) (*36*). We then probed the extent of drug effects on endogenous protein levels of GR and BMAL1 in muscle at 4 hours after a light-phase prednisone intraperitoneal pulse, challenging the interaction with overexpression of FLAG-tagged REV-ERBα. After GR-based IP, we found an enrichment for BMAL1 signal in drug- versus vehicle-injected mice (Fig. 4D), while the drug effect was blunted in the presence of REV-ERBα overexpression (Fig. 4D and inputs in fig. S4E). It must be noted that, despite the fact that REV-ERBα overexpression reduced overall BMAL1 levels as expected (*37*), the drug effects noted in this experiment matched the translocation trends found with the GFP-tagged BMAL1 construct, which escapes the transcriptional regulation by REV-ERBα. Furthermore, REV-ERBα was shown to regulate GR stability and translocation through physical interaction with the cytoplasmic docking protein HSP90 (heat shock protein 90) (*35*). We thus tested the light-phase prednisone effects on endogenous protein levels in HSP90-bound versus nuclear muscle fractions at 4 hours after pulse (ZT0) in quadriceps muscle lysates. Nuclear fractionation was checked through histone 3 enrichment (fig. S4F). In the absence of apparent changes in total protein levels (total lysate inputs in fig. S4G), the light-phase drug pulse reduced the HSP90-bound GR and BMAL1 levels, increasing their signal in the nuclear fraction, while REV-ERBα showed opposite trends (Fig. 4E). This was confirmed in myofibers electroporated with a GFP-tagged REV-ERBα construct, which showed decreased nuclear translocation in response to ex vivo drug exposure (fig. S4H). In aggregate, these data indicate that light phase–restricted prednisone promoted GR-BMAL1 interaction in muscle, and this interaction was antagonized by the BMAL1-antagonist REV-ERBα.

## DISCUSSION

### Muscle bioenergetic response to light-phase prednisone requires BMAL1

In this study, we used prednisone as exogenous glucocorticoid drug. Compared to other synthetic glucocorticoids, prednisone is ideal to assess circadian gating due to rapid uptake in striated muscles and short half-life (*21*). Prednisone was effective in unmasking the time-specific effects on muscle bioenergetics in our study. Moreover, the present data in WT and BMAL1-KO muscles compound our previous findings with pulsatile and intermittent dosing in dystrophic and injured muscles (*3*, *4*, *38*). In those prior studies, injections were performed at 7 a.m. (ZT1), a time of dose close to the ZT0 used here. The applicability of these muscle-centered findings to other synthetic glucocorticoids remains an open question. Dexamethasone elicited phase-dependent GR programs in the liver (*16*), but this is still untested in the muscle. An important consideration regarding dexamethasone is that, at least in rodents, this drug is a potent activator of wasting and muscle atrophy even at very low doses (*39*), thereby potentially overshadowing possible time-dependent effects on bioenergetics. New glucocorticoid derivatives such as vamorolone, currently in trial for Duchenne muscular dystrophy (NCT03439670), will likely offer new insights, although the time-dependent pharmacology for vamorolone will likely be very different from the mechanisms reported here as this drug minimizes the GR transactivation activity (*40*). Furthermore, here, we focused on unconventional bioenergetic pathways stimulated by exogenous prednisone through circadian time of dosing. The question of whether the GR-BMAL1-PGC1α axis can be elicited in muscle through circadian manipulation of the endogenous hypothalamic-pituitary-adrenal (HPA) axis and corticosterone fluctuations is still open and must be addressed with dedicated experiments.

Here, we reported on noncanonical, BMAL1-dependent GR effects on muscle bioenergetics triggered by exogenous glucocorticoid dosing in off-phase with endogenous glucocorticoids, i.e., in the early light period in mice. The mitochondrial stimulation of a light phase–specific prednisone pulse required muscle PGC1α expression, as shown by muscle-restricted inducible ablation of this factor. Our experiments conducted with analyses at 24 hours after pulse cannot rule out transient versus adaptive changes in muscle metabolism, although the ChIP-seq data at 4 hours after pulse indicate continuity between the acute epigenetic/transcriptional effects and the metabolic changes observed at 24 to 48 hours after pulse, as well as after 12-week-long intermittent regimens. Further studies in muscle-autonomous, time-restricted glucocorticoid effects will refine dosing and open previously unanticipated muscle-targeting indications of these widely used drugs. We anticipate that this will decrease side effect burden in long-term chronic settings and reduce—if not reverse—the dysmetabolic effects of these drugs.

Here, we focused on the direct remodeling of mitochondrial capacity in muscle through noncanonical steroid-elicited pathways. The gains in NAD^+^ and mitochondrial capacity correlated, as expected, with increased endurance and VO_2_/lean mass levels. However, we did also find light-phase prednisone effects on muscle mass and muscle force, which are generally not directly regulated by oxidative capacity. The effects we described here in WT muscle are consistent with the effects we found with intermittent prednisone (ZT1 dosing) in dystrophic muscle (*3*, *38*). The actual mechanisms mediating this “pro-ergogenic” effect (contemporaneous gain of both mass and metabolic function) are still unknown for pulsatile glucocorticoid dosing. However, muscle-specific *Nampt*-KO mice showed prominent ATP depletion accompanied by decreased force production, and this was rescued by supplementation of the NAD^+^ precursor nicotinamide riboside (*41*). In addition, it is worth noting that NAD^+^ elevation has been linked to anabolic pathways through sirtuin-based deacetylation of Forkhead Box O3 (FOXO3) for degradation (*42*), AKT for phosphatidylinositol 3,4,5-trisphosphate–induced activation (*43*), and insulin receptor substrate 2 (IRS2) for insulin-mediated activation (*44*). On the other hand, the extent to which improved bioenergetics can drive this balance is still unknown. An obvious example of this notion is exercise, which generally promotes both muscle mass and its aerobic capacity (*45*). However, in our experiments, we did not find treatment effects on spontaneous activity and locomotion in mice. This suggests that light-phase prednisone is probably stimulating the capacity of muscle to perform rather than enhancing muscle function through activity/exercise.

### Glucocorticoid “chronopharmacology” to increase muscle bioenergetics requires the activating complex of the circadian clock core

Our findings on the role of BMAL1, which is active during the light phase in mice, in pharmacological modulation of muscle mitochondria correlate well with reverse findings with factors such as Cryptochrome Circadian Regulator (CRY) proteins, which are active during the dark-phase in mice. CRY proteins suppress the transcriptional activity of CLOCK/BMAL1 complex through direct binding (*46*). CRY factors are selective repressors of peroxisome proliferator–activated receptor δ (PPARδ) and oxidative performance in muscle, as shown by double KO of both CRY1 and CRY2 in myotubes and muscle (*47*). As GR is known to directly regulate expression of the CRY cofactor PER2 through a canonical GRE site (*12*), further studies are required to disentangle the exogenous versus endogenous glucocorticoid effects on the repressive arm of the clock complex in muscle. CRY1/2 binds and suppresses GR in the dark phase in mice (*13*), and this could be an additional mechanism explaining why dark-phase prednisone did not induce the same effects observed with light-phase prednisone. In addition, the stoichiometric competition between GR and the Constitutively photomorphogenic 1 (COP1) ubiquitin machinery is critical in sequestering CRY factors for degradation (*14*). In that regard, altering the phase and/or competition of COP1-CRY binding via time-specific exogenous glucocorticoids could likely perturb the disappearance of the repressive arm of the core clock machinery. Moreover, it must be noted that CRY1 is negatively regulated by REV-ERBα and by itself (*11*). Therefore, the specific roles of CRY proteins in exogenous glucocorticoid effects according to time of dosing are likely multifaceted and must still be directly investigated. Our study did not address the circadian-independent pharmacological effects on immune system and fibrosis regulation. In the liver, glucocorticoids induce circadian-independent effects on the immune system (*15*), but this still needs to be investigated in the context of muscle remodeling.

Recently, REV-ERBα was shown to increase the propensity of liver GR to activate gene programs altering metabolism through a direct protein-protein interaction with the GR (*15*). Although still unproven in muscle, this paradigm is consistent and complementary with our findings in muscle, where an antiphysiologic steroid spike subtracts the light-phase GR from REV-ERBα, promoting GR interaction with BMAL1 and the downstream genomic effects on genes involved in mitochondrial regulation. In our experiments, the REV-ERBα effects on the GR-BMAL1 interaction may involve immediate transcriptional effects on endogenous BMAL1 and GR. However, trends confirming the antagonistic role of REV-ERBα in the drug effects on simultaneous translocation and physical interaction were also found with tagged constructs that escape the transcriptional control by endogenous or exogenous REV-ERBα.

Notably, the muscle-autonomous effects of REV-ERBα are still unclear. Initial findings in muscles from body-wide REV-ERBα-KO indicated a role in promoting muscle function (*48*). These were echoed by muscle ChIP-seq data showing REV-ERBα–suppressing muscle atrophy agonists (*19*). However, contrasting results from heterozygous versus homozygous mice for body-wide REV-ERBα-KO on muscle regeneration and growth were reported (*49*). Moreover, studies with REV-ERBα pharmacological inhibitors and REV-ERBα manipulation in isolated myoblasts indicated a negative role for this factor in muscle growth (*49*–*51*). In addition, body-wide REV-ERBα-KO was shown to ameliorate muscle pathology in dystrophic *mdx* mice (*52*). Muscle-specific, inducible manipulation of REV-ERBα will likely shed more light on its role in and beyond muscle pharmacology.

In our study, we did not find prominent changes in the period of BMAL1 reporter oscillations or in *Nr1d1* or *Per2* expression in muscle. In addition, metabolic cage and corticosterone analyses showed no prominent changes with either light-phase or dark-phase prednisone on overall circadian rhythm of WT mice. However, we recognize that the time-specific steroid effects on muscle circadian transcriptome and circadian gene oscillation were not addressed. Unbiased characterization of these transcriptional and epigenomic effects will likely shed more light on transient versus enduring changes of exogenous glucocorticoids on muscle clock regulation.

### BMAL1 and muscle PGC1α mediate the bioenergetic effects of light-phase prednisone in muscle

Our data point at BMAL1 as required mediator for light-phase prednisone effects on muscle bioenergetics after both chronic intermittent regimens and a single pulse. Here, we used whole-body BMAL1-KO mice, performing all analyses before systemic muscle wasting induced by the genotype is prominent (*22*). The multifaceted role of muscle-autonomous BMAL1 in regulating muscle bioenergetics is known (*18*, *20*, *24*, *53*). Moreover, our ChIP-seq data quantitated the drug-responsive BMAL1 epigenomic activity specifically in muscle, correlating with trends in NAD^+^ and mitochondrial respiration. The effects of light-phase prednisone on *Nampt* up-regulation and NAD^+^ biogenesis in muscle are consistent with the positive effects of NAD^+^ replenishment in normal, dystrophic, and aging muscle (*54*–*56*). Note that our findings with NAD^+^ replenishment and BMAL1 activation after light-phase prednisone are consistent also with the recently reported NAD^+^-dependent activation of BMAL1 epigenomic activity through PER2-K680 deacetylation (*57*). Furthermore, in our experiments, the trends in NAD^+^ levels correlated with treatment effects on mitochondrial performance and PGC1α deacetylation. However, we recognize that the exact NAD^+^-dependent mechanisms elicited by light-phase prednisone must still be identified. To that regard, dedicated studies on the extent to which aging and dysfunction impair the muscle response to prednisone in the light versus dark periods will provide valuable insight in translating these findings in chronic treatments for muscle conditions.

The light-phase prednisone effects on mitochondrial biogenesis and oxidative capacity in muscle were PGC1α dependent, unveiling an unprecedented relationship between this transcription factor and glucocorticoids through time-specific dosing. Our studies with the muscle-restricted PGC1α-KO muscle were performed immediately after tamoxifen induction, to avoid possible disturbances and compensatory adaptations of muscle to long-term lack of PGC1α (*58*, *59*). In addition, our data showed that tamoxifen-treated floxed^+^/Cre^−^ muscle recapitulated the effects found in WT muscle, thereby suggesting that tamoxifen did not significantly interfere with the effects elicited by prednisone chronodosing in muscle. To our knowledge, this is the first report of inducible muscle-restricted PGC1α-KO mice, and this model will be useful in unveiling PGC1α dependence of mitochondrial manipulations in postnatal muscle.

Our ChIP-seq datasets revealed epigenomic convergence of GR and BMAL1 with light-phase prednisone in muscle chromatin. Moreover, this effect was BMAL1 dependent, particularly with regard to promoters of genes with direct relevance for bioenergetics, such as *Nampt* and *Ppargc1a*. The total number of GR peaks in BMAL1-KO muscle was decreased as compared to BMAL1-WT muscle, but the relative GR signal on the GRE motif was not sizably different between the two genotypes at baseline. Moreover, in both genotypes, the number of peaks increased with the drug pulse, as compared to vehicle control, but the drug-driven gain of GR signal on GRE motif was considerably higher in BMAL1-WT than BMAL1-KO muscle. These findings indicate that BMAL1 is engaged by a light phase–restricted prednisone pulse to increase GR sensitivity to exogenous drugs during the endogenous glucocorticoid trough. Moreover, this interaction is required to stir the GR toward gene targets such as *Nampt* and *Ppargc1a*, regulating mitochondrial function. Further studies are required to define dynamics and cross-regulations at the chromatin level among these and other clock factors, particularly with regard to the emerging concept of “pioneering factors” for the CLOCK/BMAL1 complex (*60*). In this regard, it is worth emphasizing that we have focused our studies on promoter activity of GR and BMAL1 based on the region-specific enrichment data suggested by the ChIP-seq analyses. However, BMAL1 also regulates rhythmic gene programs through enhancer binding (*61*) and enhancer-enhancer interactions (*62*). Dedicated studies will be needed to evaluate this long-range BMAL1 epigenomic activity in muscle downstream of time-specific glucocorticoid stimulation.

### Limitations of mouse-to-human translation of chronopharmacology studies

Chronopharmacology studies in mice are not immediately translatable to humans because of the mismatch in circadian cycles and the complexity of feeding/activity/sleep behavior in humans (*63*). Nonetheless, the molecular oscillations governing muscle metabolism and function are conserved (*64*). Moreover, disruption of the circadian clock in muscle disrupts metabolic function in mice (*19*, *65*), aligning with the known metabolic disturbances induced by circadian disruption in humans (*8*). Thus, identifying the molecular mediators of time-specific drug dosing in murine models is still a cogent strategy to discern translatable mechanisms and biomarkers of chronopharmacology (*66*). Here, we reported epigenetic (GR-BMAL1 convergence) and metabolic (PGC1α-dependent mitochondrial regulation) determinants of light-phase glucocorticoid effects on muscle bioenergetics in mice. Further studies are required to pinpoint the relationships between steroid regimens and physiological circadian cues such as food intake and sleep/activity to better translate these findings.

In summary, our work reports molecular mechanisms and genetic mediators of bioenergetic effects in muscle triggered by an exogenous steroid pulse in the light-phase phase of mice, i.e., the phase where endogenous glucocorticoids are the lowest. The molecular and metabolic effects elicited by this chronopharmacology study will pave the way to further studies in nonmyocyte components of muscle and muscle-tailored therapies to promote the reported effects without off-target drug effects.

## MATERIALS AND METHODS

### Animal handling and treatments

Mice were housed in a pathogen-free facility in accordance with the American Veterinary Medical Association and under protocols fully approved by the Institutional Animal Care and Use Committee at Northwestern University Feinberg School of Medicine (#ISO00011692) and at Cincinnati Children’s Hospital Medical Center (#2020-0008). Consistent with the ethical approvals, all efforts were made to minimize suffering. Euthanasia was performed through carbon dioxide inhalation followed by cervical dislocation and heart removal. Mice were maintained on a 14-hour light/10-hour dark cycle, and diet/pharmacological treatments were initiated at ~12 weeks of age. Mice were obtained and interbred from the Jackson Laboratories (Bar Harbor, ME): WT C57BL/6 mice #000664, BMAL1-KO mice #009100, WT and KO littermates obtained from heterozygous parents, PGC1α-KO mice from crossing #025750 and #009666, Cre^−^ and Cre^+^ littermates obtained from Ppargc1a^fl/fl^ × Ppargc1a^fl/fl^, and HSA-CreERT2^+/−^ matings. Gene ablation was induced with tamoxifen right before start of drug treatments using a combination of intraperitoneal (20 mg/kg per day for 5 days; #T5648, Sigma-Aldrich) and chow-mediated (40 mg/kg until 48 hours before start; Harlan #TD.130860) administration (*67*). Weekly prednisone treatment consisted of once-weekly intraperitoneal injection of prednisone (1 mg/kg; #P6254, Sigma-Aldrich, St. Louis, MO) (*4*). The injectable solution was diluted from a stock (5 mg/ml) in dimethyl sulfoxide (DMSO; #D2650, Sigma-Aldrich, St. Louis, MO) in a 50-μl volume. Injections were conducted either at the beginning of the light phase (ZT0; lights on) or at the beginning of the dark phase (ZT14; lights off). Tissues were harvested 24 hours after single pulse or last injection in chronic treatment. All in vivo, ex vivo, and postmortem analyses were conducted blinded to treatment group.

### Analyses of muscle function, lean and muscle mass, and myofiber typing

Forelimb grip strength was monitored using a meter (#1027SM, Columbus Instruments, Columbus, OH) blinded to treatment groups. Animals performed 10 pulls with 5-s rest on a flat surface between pulls. Grip strength was expressed as force normalized to body weight. Running endurance was tested on a motorized treadmill with plastic nonelectrified resting posts (#1050RM, Columbus Instruments, Columbus, OH). Speed was accelerated at 1 m/min^2^ starting at 1 m/min, and individual test was interrupted when the subject spent >30 s on resting post. Running endurance was analyzed as weight normalized work (in microjoules), i.e., body weight (in grams) × distance^2^ (in square meters)/time^2^ (in square seconds).

Immediately before euthanasia, in situ tetanic force from tibialis anterior muscle was measured using the Whole Mouse Test System (catalog no. 1300A, Aurora Scientific, Aurora, ON, Canada) with a 1-N dual-action lever arm force transducer (300C-LR, Aurora Scientific, Aurora, ON, Canada) in anesthetized animals (0.8 liter/min of 1.5% isoflurane in 100% O_2_). Specifications of tetanic isometric contraction are the following: initial delay, 0.1 s; frequency, 200 Hz; pulse width, 0.5 ms; duration, 0.5 s; stimulation, 100 mA (*4*). Muscle length was adjusted to a fixed baseline of ~50-mN resting tension for all muscles/conditions. Fatigue analysis was conducted by repeating tetanic contractions every 10 s until complete exhaustion of the muscle (50 cycles). Time of contraction was assessed as time to maximal rate of force increase after initial stimulation (time to max, *dx/dt*), while time of relaxation was assessed as time to maximal rate of force decrease after tetanic stimulation cessation (time to min, *dx/dt*). Force was analyzed as specific force, i.e., maximum tetanic force normalized by average myofiber cross-sectional area. Myofiber cross-sectional areas were obtained from histology analyses at end point. Excised tissues were fixed in 10% formaldehyde (catalog no. 245-684, Thermo Fisher Scientific, Waltham, MA) at room temperature for ~24 hours and then stored at 4°C before processing. Seven-micrometer sections from the center of paraffin-embedded muscles were stained with hematoxylin and eosin (catalog no. 12013B, 1070C, Newcomer Supply, Middleton, WI). Cross-sectional area (CSA) quantitation was conducted on >400 myofibers per tissue per mouse. Imaging was performed using an Axio Observer A1 microscope (Zeiss, Oberkochen, Germany), using 10× and 20× (short-range) objectives. Images were acquired through GRYPHAX software (version 1.0.6.598; Jenoptik, Jena, Germany) and quantitated through ImageJ (*68*).

Magnetic resonance imaging (MRI) scans to determine lean mass ratios (percentage of total body mass) were conducted in nonanesthetized, nonfasted mice at ZT8 using the EchoMRI-100H Whole-Body Composition analyzer (EchoMRI, Houston, TX). Mice were weighed immediately before MRI scan. Before each measurement session, system was calibrated using the standard internal calibrator tube (canola oil). Mice were scanned in sample tubes dedicated to mice comprised between 20 and 40 g of body mass. Data were collected through built-in software EchoMRI version 140320. Data were analyzed when hydration ratio was >85%.

Muscle mass was calculated as muscle weight immediately after euthanasia and explant, normalized to tibia length. For myofiber typing, sections were incubated with primary antibodies BA-F8 (1:10), SC-71 (1:30), and BF-F3 (1:10; all by Developmental Studies Hybridoma Bank, Iowa City, IA) overnight at 4°C. Then, sections were incubated with secondary antibodies Alexa Fluor 350 anti–immunoglobulin G2b (IgG2b), Alexa Fluor 488 anti-IgG1, and Alexa Fluor 594 anti-IgM (catalog nos. A21140, A21121, and 1010111, Life Technologies, Grand Island, NY). Type 1 fibers stained blue, type 2A stained green, type 2X showed no staining, and type 2B stained red. Myofiber types were then quantitated over at least five serial sections and quantitated as the percentage of total counted myofibers. All analyses were conducted blinded to treatment.

### Metabolic cages and tissue respirometry

VO_2_ (in milliliters per hour) was assessed via indirect calorimetry using the TSE Automated Phenotyping System PhenoMaster (TSE Systems, Chesterfield, MO) at the NU Comprehensive Metabolic Core. Data collection started at 24 hours after prednisone or vehicle injection and lasted for 5 days. Results are expressed as average values (all mice per group, all values per mouse, average of 5 days) over a circadian period, as well as in an analysis of covariance (ANCOVA) (test for difference in regression lines), with average values of active phase plotted against lean mass values per mouse, as recommended by (*69*). Activity/exercise was monitored in hourly values of spontaneous free-range locomotion in the cages without addition of a wheel. Food intake was quantitated as hourly values of chow intake.

Basal tissue oxygen consumption rate (OCR) values were obtained from basal rates of oxygen consumption of muscle biopsies at the Seahorse XF96 Extracellular Flux Analyzer platform (Agilent, Santa Clara, CA) using previously detailed conditions (*4*). Basal OCR was calculated as baseline value (average of three consecutive reads) minus value after rotenone/antimycin addition (average of three consecutive reads). Basal OCR values were normalized to total protein content, assayed in each well after the Seahorse run through homogenization and Bradford assay. Nutrients were 10 mM glucose and 1 mM palmitate–bovine serum albumin (BSA; #G7021 and #P0500; MilliporeSigma, St. Louis, MO); inhibitors were 0.5 μM rotenone + 0.5 μM antimycin A (Agilent).

RCR values were obtained from isolated mitochondria from muscle tissue. Quadriceps are harvested from the mouse and cut up into very fine pieces. The minced tissue is placed in a 15-ml conical tube (#188261, USA Scientific), and 5 ml of MS-EGTA buffer [mannitol (#M0214-45, ChemProducts), sucrose (#100892, Millipore), Hepes (#15630-080, Gibco), and EGTA (#E14100-50.0, RPI)] with 1 mg of trypsin (#T1426-50MG, Sigma-Aldrich) is added to the tube. The tube is quickly vortexed, and the tissue is left submerged in the solution. After 2 min, 5 ml of MS-EGTA buffer with 0.2% BSA (#A-421-250, GoldBio) is added to the tube to stop the trypsin reaction. The tube is inverted several times to mix and then set to rest. Once the tissue has mostly settled to the bottom of the tube, 3 ml of buffer is aspirated, and the remaining solution and tissue are transferred to a 10-ml glass tissue homogenizer (#89026-382, Avantor). Once sufficiently homogenized, the solution is transferred back into the 15-ml conical tube and spun in the centrifuge at 1000*g* for 5 min at 4°C. After spinning, the supernatant is transferred to a new 15-ml conical tube. The supernatant in the new tube is then centrifuged at 12,000*g* for 10 min at 4°C to pellet the mitochondria. The supernatant is discarded from the pellet, and the pellet is then resuspended in 7 ml of MS-EGTA buffer and centrifuged again at 12,000*g* for 10 min at 4°C. After spinning, the supernatant is discarded, and the mitochondria are resuspended in 1 ml of Seahorse medium (#103335-100, Agilent) with supplemented 10 μl of 5 mM pyruvate (#P2256-100G, Sigma-Aldrich) and 10 μl of 5 mM malate (#20765, Cayman Chemical). After protein quantitation using a Bradford assay (#5000001, Bio-Rad), 2.5 μg of mitochondria are dispensed per well in 180-μl total volumes and let to equilibrate for 1 hour at 37°C. Twenty microliters of 5 mM ADP (#01905, Sigma-Aldrich), 50 μM oligomycin (#495455-10MG, Milipore), 100 μM carbonyl cyanide-*p*-trifluoromethoxyphenylhydrazone (#C3463, TCI), and 5 μM rotenone (#557368-1GM, Milipore)/antimycin A (#A674-50MG, Sigma-Aldrich) are added to drug ports A, B, C, and D, respectively, to yield final concentrations of 0.5 mM, 50 μM, 10 μM, and 0.5 μM.At baseline and after each drug injection, samples are read for three consecutive times. RCR was calculated as the ratio between state III (OCR after ADP addition) and uncoupled state IV (OCR after oligomycin addition). All metabolic cage and Seahorse measurements were conducted blinded to treatment groups.

### Unlabeled and labeled metabolite quantitation in muscle

Total hydrophilic metabolite content was extracted from quadriceps muscle tissue at treatment end point through methanol/water (80:20) extraction, adapting conditions described previously (*70*). Briefly, total metabolite content from quadriceps muscle was obtained from ~100 mg (wet weight) of quadriceps muscle tissue per animal. Frozen (−80°C) muscle was pulverized in liquid nitrogen and homogenized with ~250 μl of 2.3-mm zirconia/silica beads (catalog no. 11079125z, BioSpec, Bartlesville, OK) in 1 ml of methanol/water (80:20, v/v) by means of Mini-BeadBeater-16 (catalog no. 607, BioSpec, Bartlesville, OK) for 1 min. After centrifuging at 5000 rpm for 5 min, 200 μl of supernatant were transferred into a tube preadded with 800 μl of ice-cold methanol/water (80%, v/v). Samples were vortexed for 1 min and then centrifuged at ~20,160*g* for 15 min at 4°C. Metabolite-containing extraction solution was then dried using SpeedVac (medium power). A total of 200 μl of 50% acetonitrile were added to the tube for reconstitution, followed by overtaxing for 1 min. Samples solution were then centrifuged at 20,000*g* for 15 min at 4°C. Supernatant was collected for liquid chromatography (LC)–MS analysis for hydrophilic metabolite profiling as follows. Samples were analyzed by high-performance LC and high-resolution MS and tandem MS (HPLC-MS/MS). Specifically, system consisted of a Thermo Q-Exactive in line with an electrospray source and an UltiMate 3000 (Thermo Fisher Scientific) series HPLC consisting of a binary pump, degasser, and autosampler outfitted with an Xbridge Amide column (Waters; dimensions of 4.6 mm by 100 mm and a 3.5-μm particle size). The mobile phase A contained 95% (v/v) water, 5% (v/v) acetonitrile, 20 mM ammonium hydroxide, and 20 mM ammonium acetate (pH 9.0); the mobile phase B was 100% acetonitrile. The gradient was as follows: 0 min, 15% A; 2.5 min, 30% A; 7 min, 43% A; 16 min, 62% A; 16.1 to 18 min, 75% A; 18 to 25 min, 15% A with a flow rate of 400 μl/min. The capillary of the electrospray ionization source was set to 275°C, with sheath gas at 45 arbitrary units, auxiliary gas at 5 arbitrary units, and the spray voltage at 4.0 kV. In positive/negative polarity switching mode, a mass/charge ratio scan range from 70 to 850 was chosen, and MS1 data were collected at a resolution of 70,000. The automatic gain control target was set at 1 × 10^6^, and the maximum injection time was 200 ms. The top five precursor ions were subsequently fragmented, in a data-dependent manner, using the higher-energy collisional dissociation cell set to 30% normalized collision energy in MS2 at a resolution power of 17,500. The sample volumes of 25 μl were injected. Data acquisition and analysis were carried out by Xcalibur 4.0 software and TraceFinder 2.1 software, respectively (both from Thermo Fisher Scientific). Metabolite levels were analyzed as peak area normalized to wet tissue weight (weight before cryopulverization). Metabolite analysis was performed blinded to treatment groups.

^13^C tracing from nutrients in muscle was performed adapting reported conditions (*71*) to our muscle stimulus settings used to probe muscle force (see below). Immediately after euthanasia, quadriceps muscles were dissected and immobilized on a Sylgard-coated well of a 12-multiwell plate by means of two 27-gauge needles at the muscle extremities. The well was prefilled with 1× Ringers’ solution [146 mM NaCl, 5 mM KCl, 2 mM CaCl_2_, 1 mM MgCl_2_, and 10 mM Hepes (pH 7.4)] containing insulin (25 mU/ml; catalog no. RP-10908, Thermo Fisher Scientific, Waltham, MA) and the appropriate ^13^C nutrient and kept at 37°C on a heated pad. ^13^C-labeled nutrients were 10 mM 1,2-^13^C_2_-glucose and 1 mM 1-^13^C-palmitate (BSA conjugated) (#453188 and #292125,Sigma-Aldrich). The nutrient solution was constantly bubbled with a 95% O_2_/5% CO_2_ line (~2 psi). After 5-min equilibration in solution, electrodes were inserted at the muscle extremities, securing them to the holder needles. Using the Whole-Mouse Test System (catalog no. 1300A, Aurora Scientific, Aurora, ON, Canada), 20 contractions (1×/min) were induced with following specifications: initial delay, 0.1 s; frequency, 200 Hz; pulse width, 0.5 ms; duration, 0.5 s; stimulation, 100 mA. Muscles were then removed from the ^13^C nutrient solution, quickly rinsed in nutrient-free Ringers’ solution, dried, and immediately flash-frozen. Muscle metabolites were then extracted and analyzed as per metabolomics procedures (LC-MS, see above), and mass resolution was carried on predetermined metabolites, while control energetics (ATP and phosphocreatine) were analyzed from simultaneous quantitation from the LC-MS system. Metabolite labeling ratio was calculated on peak area per milligram of tissue values subtracting the background ^13^C labeling ratio obtained from muscles exposed to unlabeled nutrients (same reagents used for respirometry) and expressed as the percentage of total metabolite. Metabolite analysis was performed blinded to treatment groups.

### Mitochondrial density and NAD^+^-ATP–targeted assays

The mtDNA/nDNA assay was performed on genomic DNA isolated using the G-Biosciences Omniprep Kit (#786-136, G-Biosciences). The ratio was obtained from qPCR values (absolute expression normalized to internal standard Rn45s and DNA concentration) of NADH dehydrogenase 1 (ND1) (mtDNA locus; primers, CTAGCAGAAACAAACCGGGC, CCGGCTGCGTATTCTACGTT) versus Hexokinase 2 (HK2) (nDNA locus; primers, GCCAGCCTCTCCTGATTTTAGTGT and GGGAACACAAAAGACCTCTTCTGG) (*72*). For the MitoTracker assay, MitoTracker Green FM powder (#M7514, Invitrogen) is resuspended in 373 μl of DMSO (#BP231-100, Thermo Fisher Scientific) to obtain a 200 μM concentration. One microliter of this resuspension is added to 1 ml of Mammalian Ringer’s solution (#11763-10, Electron Microscopy Sciences) containing isolated myofibers from the FDB muscle of the mouse foot. The solution containing myofibers and MitoTracker is then pipetted into a 96-well plate (#9017, Corning) in increments of 200 μl. This plate is then read at the plate reader for fluorescence with excitation set to 490 nm and emission set to 516 nm. Values are then normalized to protein content, assayed in each well after the MitoTracker assay through homogenization and Bradford assay. NAD^+^-ATP–targeted assays were performed on ~20 mg of cryopulverized muscle tissue (per assay) using dedicated assays: a colorimetric assay for NAD [nicotinamide adenine dinucleotide (oxidized form)] (#600480, Cayman Chemical) and a luminometric assay for ATP (#700410, Cayman Chemical); both then performed using the BioTek Synergy H1 Microplate Reader.

### Chromatin immunoprecipitation sequencing

Whole quadriceps muscles (both per mouse) were cryopowdered using a liquid nitrogen–cooled RETSCH CryoMill. The cryopowdered tissue was then fixed in 10 ml of 1% paraformaldehyde (PFA) for 30 min at room temperature with gentle nutation. Fixation was quenched 1 ml of 1.375 M glycine (catalog no. BP381-5, Thermo Fisher Scientific, Waltham, MA) with gentle nutation for 5 min at room temperature. After centrifugation at 3000*g* for 5 min at 4°C, the pellet was resuspended in cell lysis buffer as per reported conditions (*73*), supplementing the cell lysis buffer with cytochalasin B (3 μg/ml) and rotating for 10 min at 4°C. Nuclei were pelleted at 300*g* for 10 min at 4°C and subsequently processed following the reported protocol with the adjustment of adding cytochalasin B (3 μg/ml) into all solutions for chromatin preparation and sonication, antibody incubation, and wash steps. Chromatin was then sonicated for 15 cycles (30 s, high power, 30-s pause, and 500-μl volume) in a water bath sonicator set at 4°C (Bioruptor 300. Diagenode, Denville, NJ). After centrifuging at 10,000*g* for 10 min at 4°C, sheared chromatin was checked on agarose gel for a shear band comprised between ~150 and ~600 bp. Two micrograms of chromatin was kept for pooled input controls, whereas ~50 μg of chromatin was used for each pull-down reaction in a final volume of 2 ml rotating at 4°C overnight. Primary antibodies were as follows: rabbit polyclonal anti-GR (#A2164, ABclonal), rabbit polyclonal anti–RNApol-II (A11181), and mouse monoclonal anti-BMAL1 (#PCRP-ARNTL-1C12, Developmental Studies Hybridoma Bank) (*74*). Chromatin complexes were precipitated with 100 μl of Dynabeads M-280 (sheep anti-mouse, #11202D; sheep anti-rabbit, #11204; Thermo Fisher Scientific, Waltham, MA). After washes and elution, samples were treated with proteinase K (catalog no. #19131, QIAGEN, Hilden, Germany) at 55°C, and cross-linking was reversed through overnight incubation at 65°C. DNA was purified using a MinElute purification kit (catalog no. 28004, QIAGEN, Hilden, Germany) and quantitated using Qubit reader and reagents. Library preparation and sequencing were conducted at the NU Genomics Core, using TrueSeq ChIP-seq library prep (with size exclusion) on ~10 ng of chromatin per ChIP sample or pooled inputs and HiSeq 50-bp single-read sequencing (~60 million read coverage per sample). Peak analysis was conducted using HOMER software (v4.10) (*75*) after aligning fastq files to the mm10 mouse genome using bowtie2 (*76*). A list of commands used is provided as the Supplementary Materials. PCA was conducted using ClustVis (*77*). Heatmaps of peak density were imaged with TreeView3 (*78*). Peak tracks were imaged through WashU epigenome browser. Gene ontology pathway enrichment was conducted using the gene ontology analysis tool (*79*).

### Live three-dimensional confocal imaging and immunofluorescence staining

GFP-tagged constructs were obtained from Origene. FDB fibers were transfected by in vivo electroporation. Methods were described previously (*80*). Briefly, the hindlimb footpad was injected with 10 μl of hyaluronidase (8 U) (catalog no. H4272, Sigma-Aldrich, St. Louis, MO). After 2 hours, up to 40 μg in 20 μl of endotoxin-free plasmid was injected into the footpad. Electroporation was conducted by applying 20 pulses, 20 ms in duration/each, at 1 Hz, at 100 V/cm. Myofibers were isolated at 7 days after transfection through collagenase II (#17101-015, Thermo Fisher Scientific) incubation and manual pipetting as previously described (*80*). Imaging was carried at room temperature using a Nikon A1R laser scanning confocal equipped with GaSP detectors through a 60× Plan Apo water immersion objective. z-stacks (10 μm in thickness, 40 slices, one slice every 125 nm) at 0 and 20 min after drug supplementation (prednisone, 25 μg/ml) were captured through the Nikon Elements AR software with fixed laser power and gain (4′,6-diamidino-2-phenylindole, 0.5 power, 10 gain; GFP, 5 power, 20 gain). Nuclear GFP signal was then quantitated using ImageJ software version 2.1.0 (*68*). Merges were obtained with regular stack merge. Nuclear GFP was quantitated using the colocalization quantitation (GFP as channel 0; Hoechst as channel 1; zero-zero included), selecting, and multimeasuring multiple regions of interest for all nuclei in the colocalized image [split channel; the colocalization channel (marked as “RED”) was considered]. Nuclear GFP intensity values were then normalized to volume and to volume-normalized cytoplasm fluorescence intensity. Three-dimensional (3D) images were rendered with the 3D Viewer plugin in ImageJ. 3D rendering of Hoechst-filtered nuclear GFP signal was obtained from the RED colocalization channel.

Immunofluorescence staining was performed on isolated myofibers using the following conditions: 4% PFA fixation (10 min at room temperature); permeabilization with 0.2% Triton X-100 (catalog no. X-100, Sigma-Aldrich, St. Louis, MO), 1% BSA (catalog no. A7906, Sigma-Aldrich, St. Louis, MO), and phosphate-buffered saline (PBS; 30 min at room temperature); blocking in 1% BSA, 10% fetal bovine serum, and PBS (30 min at room temperature); incubation at 4°C overnight with primary antibody: rabbit polyclonal anti-GR (1:100; #A2164, ABclonal) and rabbit polyclonal anti-BMAL1 (1:100; #A4714, ABclonal); and counterstaining with donkey anti-rabbit IgG Alexa Fluor 488 (1:500; #A-21206, Thermo Fisher Scientific) and Hoechst (0.5 μg/ml) at room temperature for 1 hour. Imaging was performed using a Nikon Eclipse Ti_U inverted microscope, using a 40× objective. Quantitation of myonuclear and cytoplasmic signals was performed averaging the values of >30 myonuclei (at least three myonuclei per myofiber; 10 myofibers) per mouse and was performed using ImageJ (National Institutes of Health). Imaging and analyses were conducted blinded to construct identity and treatment.

### Western blotting, IP, qPCR, corticosterone, and luciferase assays

Protein analysis was performed on ~50 μg of total lysates from whole quadriceps muscles homogenized in general protein buffer, i.e., PBS supplemented with 1 mM CaCl_2_, 1 mM MgCl_2_ (#C1016 and #M8266, Sigma-Aldrich, St. Louis, MO), and protease and phosphatase inhibitors (#04693232001 and #04906837001, Roche, Basel, Switzerland). Blocking and stripping solutions were StartingBlock and RestorePLUS buffers (#37543 and #46430, Thermo Fisher Scientific, Waltham, MA). Co-IP was performed from whole lysates of electroporated muscles using GFP- and FLAG (DDK)–tagged constructs from Origene or from whole lysates of muscles from mice after drug treatments. A total of ~100 μg of protein lysates were rotated overnight at 4°C in a final volume of 500 μl of protein buffer with 5 μl of primary antibody for pull-down. The day after, 50 μl of Dynabeads were added to the samples, with additional rotating incubation at 4°C for 4 hours. After four washes at the magnet separator, proteins were extracted from Dynabeads through incubation at 95°C for 15 min in Laemmli buffer. Input controls are 10 μg of the input protein lysates. Primary antibodies (all diluted 1:1000 for overnight incubation at 4°C) were as follows: rabbit anti-NAMPT (#A0256, ABclonal), rabbit anti-PGC1a (#A12348, ABclonal), mouse antisarcomeric α-actinin (#A7732, Sigma-Aldrich), rabbit anti–ac-Lys (#2391, ABclonal), rabbit anti–p-Ser (#AP0932, ABclonal), rabbit anti-BMAL1 (A17334), rabbit anti-GR (#A2164, ABclonal), mouse anti-FLAG (also called DDK; #TA50011, Origene), rabbit anti–REV-ERBa (#A20452, ABclonal), rabbit anti-H3 (#A2348, ABclonal), and rabbit anti-HSP90 (#A5027, ABclonal). Secondary antibody (diluted 1:5000 for 1-hour incubation at room temperature) was as follows: horseradish peroxidase–conjugated donkey anti-rabbit or anti-mouse (#sc-2313 and #sc-2314, Santa Cruz Biotechnology, Dallas, TX). Counterstain for loading control was performed with ponceau (#P7170, Sigma-Aldrich, St. Louis, MO). Blots were developed with SuperSignal Pico (catalog no. 34579, Thermo Fisher Scientific, Waltham, MA) using a iBrightCL1000 developer system (catalog no. A32749, Thermo Fisher Scientific, Waltham, MA) with automatic exposure settings. Western blotting gels and membranes were run/transferred in parallel and/or stripped for multiple antibody-based staining for densitometry analyses. Protein density was analyzed using the gel analysis tool in ImageJ software (*68*) and expressed as fold changes to control samples.

For reverse transcription qPCR (RT-qPCR) assays, total RNA was extracted from cryopulverized quadriceps muscles with TRIzol (#15596026, Thermo Fisher Scientific, Waltham, MA), and 1 μg of RNA was reverse-transcribed using 1× qScript Supermix (#95048, QuantaBio, Beverly, MA). RT-qPCR assays were conducted in triplicates using 1× Sybr Green Fast qPCR mix (#RK21200, ABclonal, Woburn, MA) and 100 nM primers at a CFX96 qPCR machine (Bio-Rad, Hercules, CA; thermal profile: 95°C for 15 s and 60°C for 30 s; 40×; melting curve). Primers were selected among validated primer sets from the MGH PrimerBank (IDs: 10946948a1, 6679433a1, 21703866a1, and 6755028a1).

The BMAL1-responsive luciferase reporter plasmid (*34*) was obtained from Addgene (#48747) and electroporated in myofibers as aforementioned for GFP-tagged vectors and following reported methods (*80*). For long-term follow up of ex vivo luciferase activity, electroporated muscles were immediately lysed and processed using protocols and reagents from the Dual Luciferase Assay Kit (catalog no. 1910, Promega, Madison, WI). Luminescence was recorded at a Synergy HTX multimode 96-well plate reader. Raw values were normalized to Renilla luciferase and to vehicle controls. For the short-term analysis of circadian fluctuations of luminescence, whole electroporated muscles were assayed ex vivo in a lumicycle (Actimetrics) without synchronization. Assay conditions were as previously described (*34*). Briefly, the full-length FDB muscle was spread flat on a 0.2-μm filter (Millipore) exposed to luciferin-containing media [1.2 ml of Dulbecco’s modified Eagle’s medium (Gibco) containing sodium bicarbonate (352.5 μg/ml), 10 mM Hepes (Gibco), 2 mM l-glutamine, 2% B-27 serum-free supplement (Invitrogen), penicillin (25 U/ml), streptomycin (20 μg/ml; Gibco), and 0.1 mM luciferin sodium salt (Biosynth AG)] on the basal side. Dishes were sealed with vacuum grease and a round cover slip and maintained in a lumicycle at 37°C. Amplitude was determined by calculating the half difference in height of the background-subtracted maxima and minima on the third phase of oscillation (~3 days after start of recordings).

For ChIP-qPCR analyses, muscle chromatin was immunoprecipitated following the conditions for ChIP-seq. Once isolated and purified after IP for GR or BMAL1, input and IP chromatin were diluted 100× and assayed using the qPCR conditions. The regions identified by peak analysis were chr5:51553620 to 51554360 for the *Ppargc1a* promoter and chr12:32819174 to 32820361 for the *Nampt* promoter. Primers were as follows: *Ppargc1a* promoter, 5′-acatgtcccaagccatccag-3′ (forward) and 5′-gctgagtctggggctacttg-3′ (reverse); *Nampt* promoter, 5′-acaggctcatggaagttggg-3′ (forward) and 5′-caacccggaccttcctcttg-3′ (reverse). Signal in IP chromatin was quantitated as percentage of input signal. Plasma corticosterone was measured using a corticosterone enzyme-linked immunosorbent assay kit (#501320, Cayman Chemical) according to the manufacturer’s instructions and internal standards to calculate values in nanograms per milliliter.

### Statistics

Statistical analyses were performed using Prism software v9.2.0 (GraphPad, La Jolla, CA). The Pearson-D’Agostino normality test was used to assess data distribution normality. When comparing two groups, two-tailed Student’s *t* test with Welch’s correction (unequal variances) was used. When comparing three groups of data for one variable, one-way analysis of variance (ANOVA) with Sidak multicomparison was used. When comparing data groups for more than one related variable, two-way ANOVA was used with Sidak multicomparison. For ANOVA and *t* test analyses, a *P* value of less than 0.05 was considered significant. When the number of data points was less than 10, data were presented as single values (dot plots and histograms). Tukey distribution bars or violin plots were used to emphasize data range distribution in analyses pooling larger data point sets per group (typically >10 data points). For curves, the SEM values for each plotted point were reported as upper and lower lines.

## Supplementary Materials

### This PDF file includes:

Figs. S1 to S4 List of commands for ChIP-seq analysis

## Notes

View/request a protocol for this paper from *Bio-protocol*.
