# Supplementary material for: Muscle mitochondrial remodeling by intermittent glucocorticoid drugs requires an intact circadian clock and muscle PGC1α
Source: Sci Adv. 2022 Feb 18;8(7):eabm1189. doi: 10.1126/sciadv.abm1189 (PMC8856622; doi:10.1126/sciadv.abm1189)
Supplement: Supplementary file 1 — Figs. S1 to S4 List of commands for ChIP-seq analysis [file sciadv.abm1189_sm.pdf]

Science Advances
AAAS

Supplementary Materials for

Muscle mitochondrial remodeling by intermittent glucocorticoid drugs requires an intact circadian clock and muscle PGC1α

Mattia Quattrocelli*, Michelle Wintzinger, Karen Miz, Daniel C. Levine, Clara Bien Peek, Joseph Bass, Elizabeth M. McNally

*Corresponding author. Email: mattia.quattrocelli@cchmc.org

Published 18 February 2022, *Sci. Adv.* **8**, eabm1189 (2022)
DOI: 10.1126/sciadv.abm1189

The PDF file includes:

Figs. S1 to S4
Legend for table S1
List of commands for ChIP-seq analysis

Other Supplementary Material for this manuscript includes the following:

Table S1

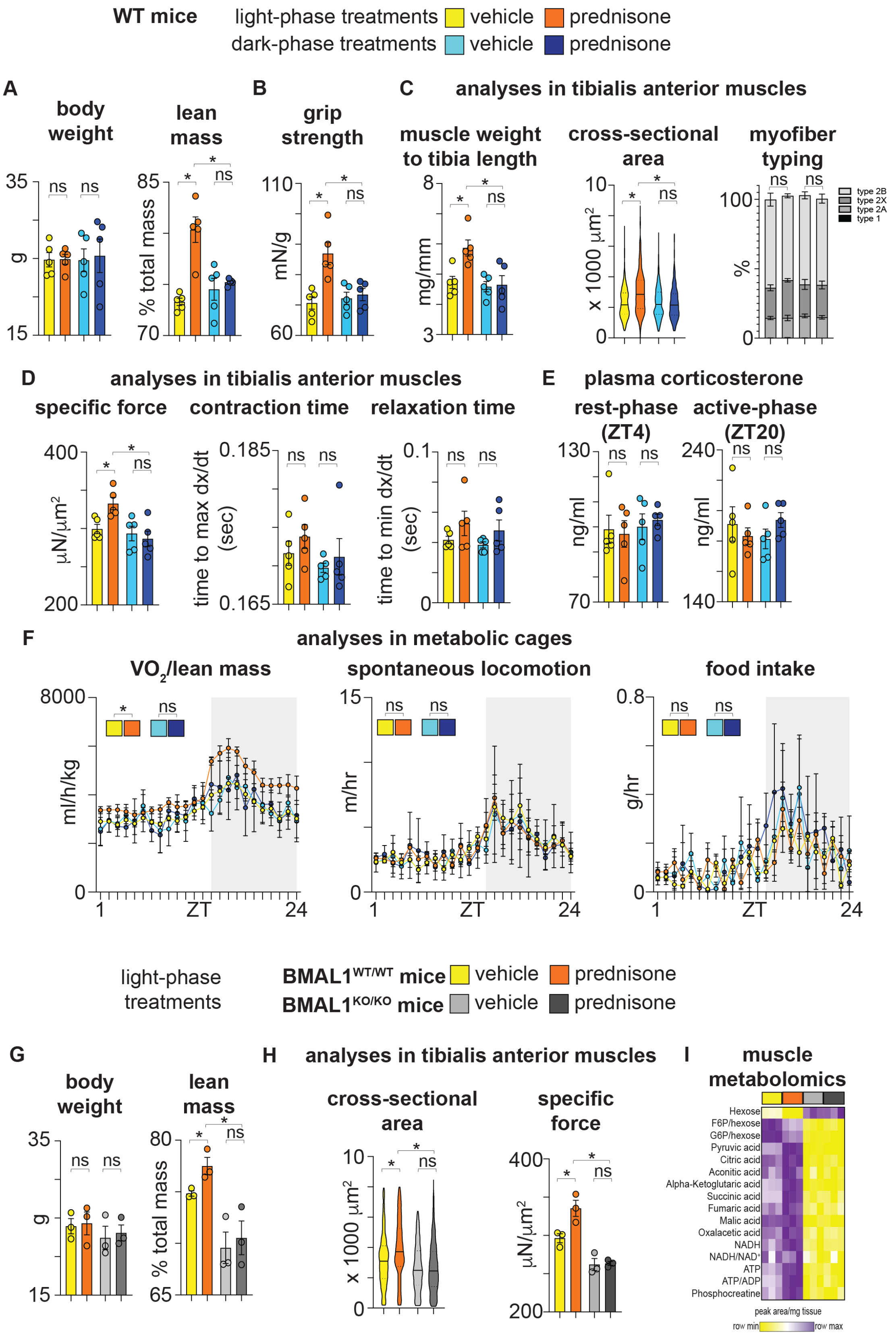
WT mice
light-phase treatments
vehicle
prednisone
dark-phase treatments
vehicle
prednisone
A
body weight
lean mass
B
grip strength
C
analyses in tibialis anterior muscles
muscle weight to tibia length
cross-sectional area
myofiber typing
type 2B
type 2X
type 2A
type 1
g
% total mass
mN/g
mg/mm
x 1000 μm²
%
D
analyses in tibialis anterior muscles
specific force
contraction time
relaxation time
μN/μm²
time to max dx/dt (sec)
time to min dx/dt (sec)
E
plasma corticosterone
rest-phase (ZT4)
active-phase (ZT20)
ng/ml
F
analyses in metabolic cages
VO2/lean mass
spontaneous locomotion
food intake
ml/h/kg
m/hr
g/hr
ZT
light-phase treatments
BMAL1WT/WT mice
vehicle
prednisone
BMAL1KO/KO mice
vehicle
prednisone
G
body weight
lean mass
H
analyses in tibialis anterior muscles
cross-sectional area
specific force
I
muscle metabolomics
Hexose
F6P/hexose
G6P/hexose
Pyruvic acid
Citric acid
Aconitic acid
Alpha-Ketoglutaric acid
Succinic acid
Fumaric acid
Malic acid
Oxalacetic acid
NADH
NADH/NAD+
ATP
ATP/ADP
Phosphocreatine
peak area/mg tissue
row min
row max

Figure S1. Additional analyses related to light-phase versus dark-phase intermittent prednisone regimens and BMAL1 requirement for light-phase prednisone effects. Results are shown after a 12-week-long treatment with intermittent once-weekly prednisone with dosing restricted to ZT0 (light-phase) versus ZT14 (dark-phase). **A)** In WT mice, ZT0 but not ZT14 prednisone increased lean mass. No changes in body weight were recorded between cohorts. **B)** ZT0 but not ZT14 prednisone increased grip strength normalized to body weight. **C-D)** In *tibialis anterior* muscles, ZT0 but not ZT14 prednisone increased muscle weight normalized to tibia length and myofiber cross-sectional area. No significant changes in myofiber typing were noted through immunostaining. In the same muscles, ZT0 but not ZT14 prednisone increased muscle force with no significant changes in contraction or relaxation times, as quantitated through in situ mechanics (Aurora) with isometric tetanic contractions. **E)** Intermittent prednisone treatments, either with ZT0 or ZT14 dosing, did not significantly affect plasma corticosterone levels in the rest-phase (trough) and active-phase (peak) at 24-hours after last injection. **F)** ZT0 but not ZT14 prednisone increased VO2 over lean mass in mice, here shown as average curves of all mice (5-day average per mouse) throughout a circadian period. Treatments did not significantly shift magnitude or timing of spontaneous locomotion or food intake, as quantitated through the metabolic cage system. **G-H)** BMAL1-KO mice blocked the ZT0 prednisone effects on body-wide lean mass, and on cross-sectional area and force of tibialis anterior muscles seen in BMAL1-WT mice. **I)** Mass-spec untargeted profiling of hydrophilic muscle metabolites showed gain of pyruvate. TCA cycle intermediates, ATP and phosphocreatine with light-phase intermittent prednisone regimen in muscle of BMAL1-WT mice. The drug effects were blunted in muscle from BMAL1-KO mice. BMAL1-KO muscles showed an overall reduction in those metabolites with an increase in hexose, in line with impaired metabolism and function in these mice. N=5(♂, ♀)/group for A-D; 3(♂, ♀)/group for E-I. *, $P<0.05$, 1w ANOVA + Sidak (histograms), 2w ANOVA (curves, stacked histogram).

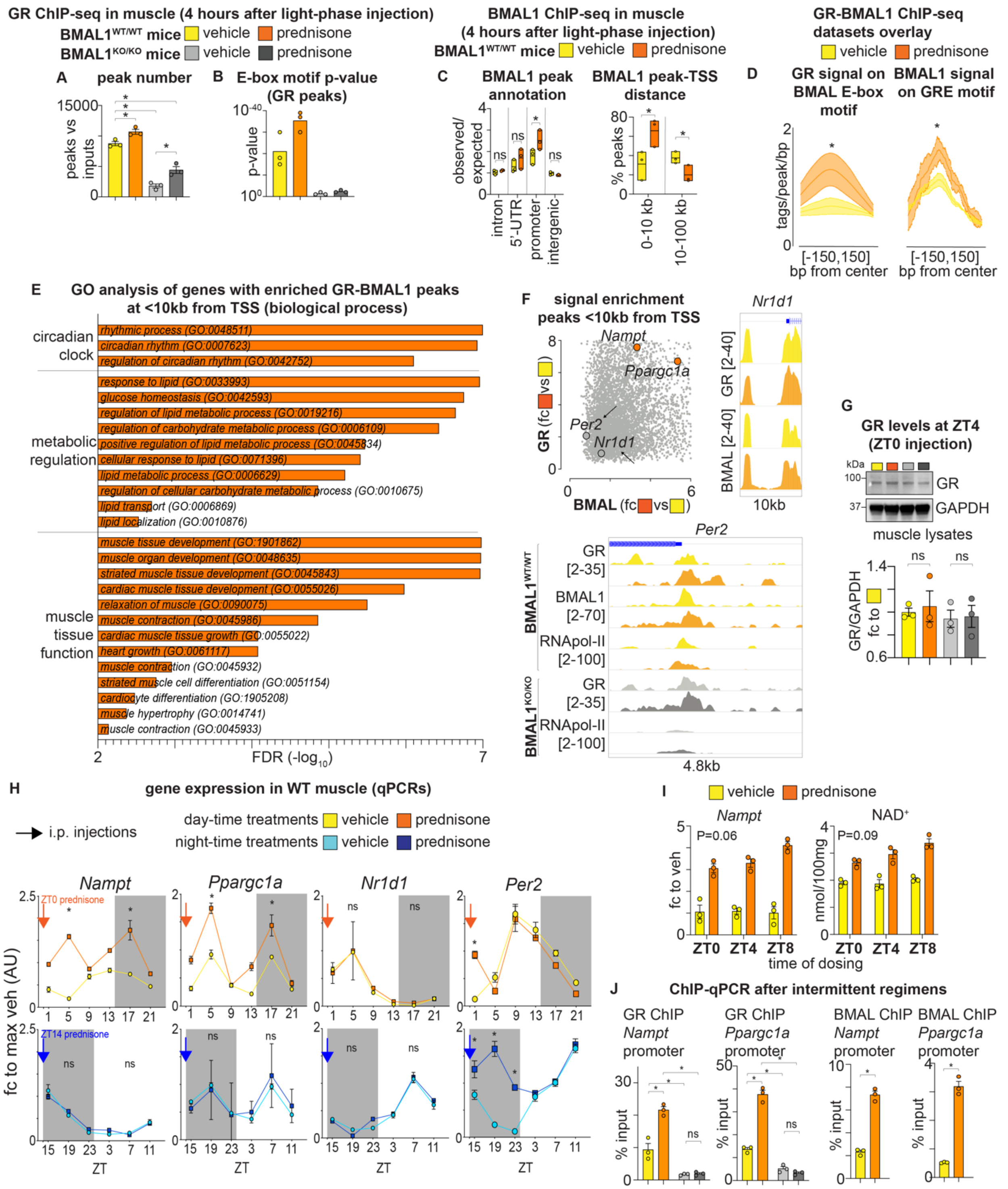

Figure S2. Additional analyses related to GR-BMAL1 epigenomic programs in muscle with a light-phase prednisone pulse. Results are shown at 4-hours after a single prednisone pulse in vivo at ZT0 (light-phase). **A-B)** Compared to BMAL1-WT muscles, BMAL1-KO muscles showed a decrease in total number of GR peaks with both vehicle and prednisone. Indeed, in unbiased motif analyses the E-box motif appeared significantly enriched in BMAL1-WT (particularly after prednisone pulse) but not in BMAL1-KO muscle. **C)** Light-phase prednisone

shifted BMAL1 peaks in muscle toward a more proximal distance to gene TSSs (<10kb), correlating with enriched peak number in annotated promoters. **D)** Light-phase prednisone increased BMAL1 occupancy on GRE-containing regions and GR occupancy on E-box-containing regions. **E)** GO terms regarding circadian clock, metabolic function and muscle regulation were enriched in the genes with gain of both GR and BMAL1 peak signal in their promoters. **F)** Unlike *Nampt* and *Ppargc1a*, clock factors like the BMAL1 target *Nr1d1* (encoding REV-ERBα) and the GR-BMAL1 target *Per2* showed variable responses to the ZT0 prednisone pulse. *Nr1d1* showed drug-unresponsive peaks of GR and BMAL1 in its proximal and distal promoter regions. *Per2* promoter showed a ~2-fold increase in GR occupancy after drug pulse, but no changes in BMAL1 binding. BMAL1-KO did not change GR enrichment but reduced RNApol-II recruitment to its TSS. **G)** No changes were noted in total GR levels in *quadriceps* muscles of BMAL1-WT and BMAL1-KO mice at ZT4 after ZT0 prednisone/vehicle injection. **H)** We monitored gene expression in muscle through qPCR every 4 hours for a circadian period after a single prednisone pulse in vivo in WT mice, comparing a pulse at ZT0 versus ZT14 (arrows). Compared to isochronic vehicle, we found that a pulse of light-phase but not dark-phase prednisone increased *Nampt* and *Pparcg1a* levels during the same phase of injection and the following phase. *Nr1d1* expression levels did not change in muscle after either light-phase or dark-phase pulses. Intriguingly, *Per2* upregulation by prednisone was much shorter-lived with ZT0 than with ZT14 prednisone. **I)** Comparing light-phase prednisone injections at ZT0-4-8 (peak of BMAL1 activity), we found a non-significant trend in enhanced *Nampt* and NAD^+ upregulation in muscle at 24-hours post-pulse. **J)** ChIP-qPCR for GR and BMAL1 targeting the peak-enriched promoter regions highlighted in Figure 2H confirmed the epigenetic effects of ZT0 prednisone after 12-week-long intermittent regimens (analyses at 4-hours after last injection). N=3♂/group (A-H), 3(♂, ♀)/group (I). *, P<0.05, 1w ANOVA + Sidak (A, I), 2w ANOVA (H), 2w ANOVA + Sidak (C, D, G).

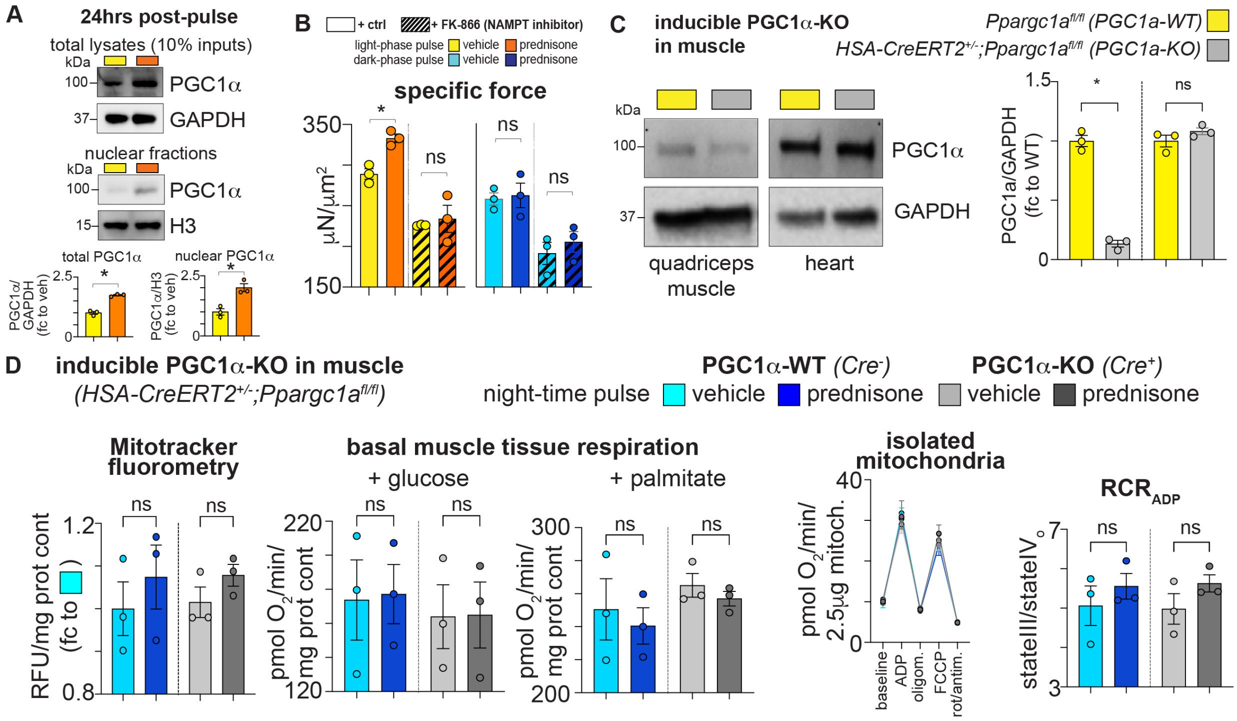

Figure S3. Additional analyses related to PGC1α levels, FK866 (NAMPT activity inhibition) and inducible PGC1α-KO muscles. A) At 24-hours after ZT0 prednisone injection, the increase in PGC1α total levels was mirrored by increase in nuclear PGC1α levels in *quadriceps* muscle, suggesting increased activity. **B)** Co-injection with the NAMPT inhibitor FK-866 blunted the ZT0 prednisone effects on muscle force. **C)** After tamoxifen injections and two weeks on tamoxifen chow, PGC1α levels in muscle were ~85% ablated, whereas no changes were seen in heart PGC1α levels. **D)** No changes were seen between PGC1α-WT and PGC1α-KO muscles after dark-phase prednisone pulse. N=3(♂, ♀)/group per time point. *, P<0.05, Welch's t-test for A, 1w ANOVA + Sidak for B-D.

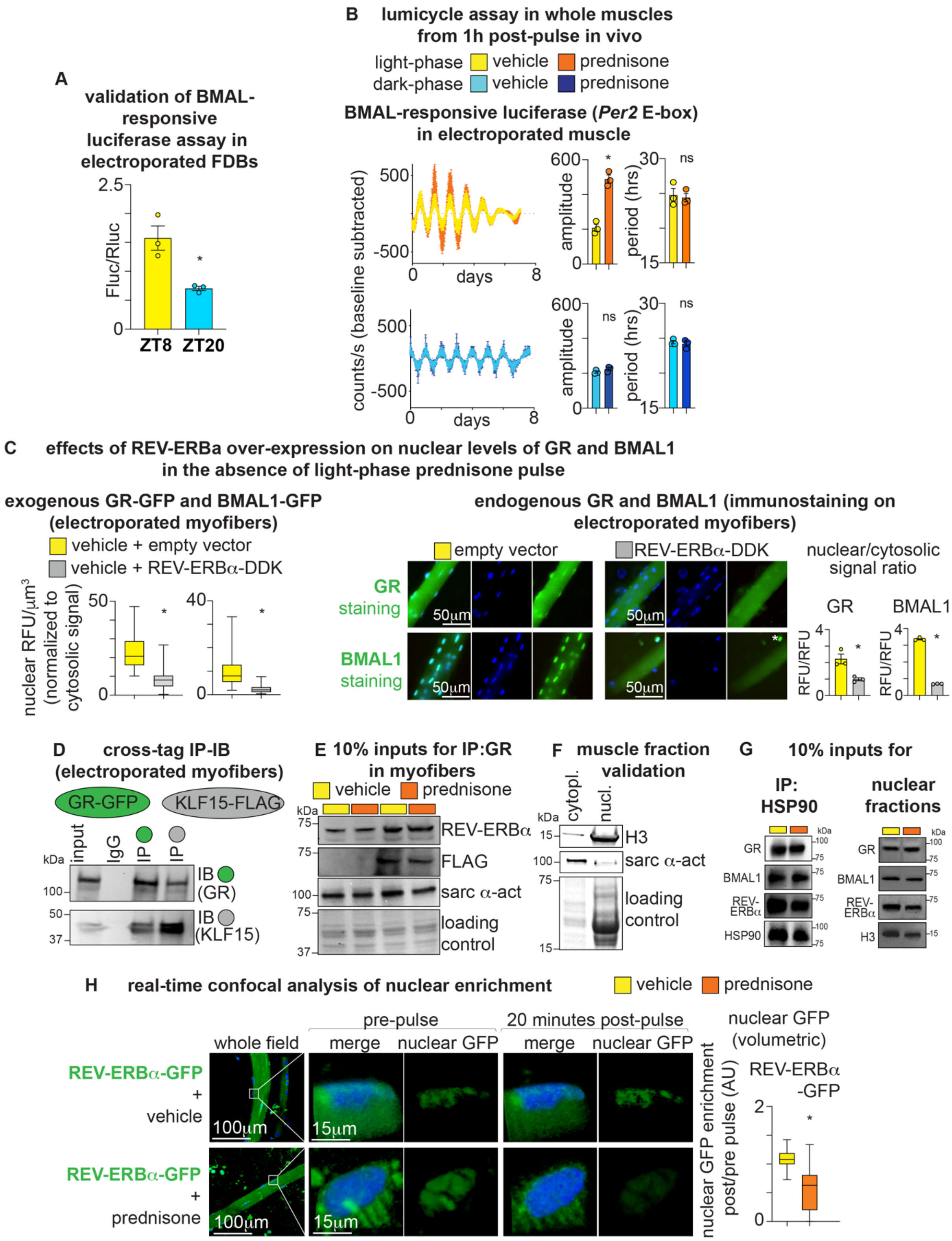

Figure S4. Additional analyses related to light-phase prednisone effects on GR-clock interactions in muscle. A) The BMAL1-responsive luciferase assay in electroporated FDB

myofibers showed higher luminescence at ZT8 than ZT20 in the absence of prednisone, following the endogenous BMAL1 activity levels. **B)** Lumicycle assay of electroporated muscles, isolated right after in vivo drug pulses, showed a transient increase in amplitude of BMAL1-responsive luciferase oscillations after light-phase prednisone. Conversely, in vivo dark-phase prednisone did not elicit this gain. Period of oscillations did not change. **C)** In the absence of prednisone stimulation, REV-ERBα overexpression decreased the myonuclear localization of exogenous GFP-tagged GR and BMAL1 in electroporated myofibers. The same trends were found through staining of endogenous GR and BMAL1 in myofibers electroporated with REV-ERBα-overexpressing versus empty vector control. *, nucleus outside myofiber. Analyses at ZT0. **D)** Cross-tag control immunoprecipitation for GR-KLF15 interaction with the same vectors used for imaging. **E)** Input muscle lysates for GR-IP experiment shown in main figure 4. **F)** Validation of nuclear fraction extraction from muscle tissue. **G)** Input lysates for IP:HSP90 and nuclear fractions shown in main Figure 4. The total muscle lysates did not show significant changes in total protein levels at 4-hours (ZT4) after a ZT0 prednisone pulse. **H)** ZT0 prednisone pulse ex vivo decreased the nuclear-confined REV-ERBα signal in electroporated myofibers. N=3(♂, ♀)/group per time point. *, $P<0.05$, Welch's t-test.

Supplementary Table 1

This table is supplemented as CSV file.

Supplementary Table 1. List of genes with prednisone versus vehicle fold change values for RNApol2 signal on TSS and BMAL1 and GR peak signal on promoter regions.

List of commands used for ChIP-seq analysis

Purpose	Command	Notes
Alignment	./bowtie2 -p 16 -x mm10 xyz.fastq > xyz.sam	
Tag Directory	makeTagDirectory xyz/ xyz.sam	
Peak track	makeUCSCfile xyz/ -o auto	
Peak Track conversion to BigWig	./bedGraphToBigWig.dms xyz.bedgraph mm10.chrom.sizes xyz.bw	
Peak finding	findPeaks xyz/ -style factor -i xyz_input/ > xyzpeaks	
Merge peaks	mergePeaks -d given xyzpeaks1 xyzpeaks2 > xyzmerged	
Motif analysis	findMotifsGenome.pl xyzpeaks mm10 xyzmotifs/ -size given	
Peak signal over motif heatmap	annotatePeaks.pl xyzpeaks mm10 -size 150 -center xyz.motif -d xyz/ xyz2/ … > xyzcenteredpeaks<br>annotatePeaks.pl xyzcenteredpeaks mm10 -size 300 -hist 1 -ghist -d xyz/ xyz2/ … > xyzheatmap	
Motif density curve	annotatePeaks.pl xyzcenteredpeaks mm10 -size 300 -hist 1 -d xyz/ xyz2/ … > xyzdensity	
Differential peak signal	getDifferentialPeaks xyzpeaks xyz/ xyz2/ … -size 300 > xyzdiffpeaks	
To define peak-TSS distance and region annotation	annotatePeaks.pl xyzpeaks mm10 -size 200 -d xyz/ -annStats xyzannStats > xyz_annotated_stats	
relative distances between GR and BMAL1 sites in differential peaks	mergePeaks xyzpeaks1 xyzpeaks2 -d 0 -matrix xyz_overlap_0bp<br>mergePeaks xyzpeaks1 xyzpeaks2 -d 100 -matrix xyz_overlap_100bp<br>…	This was repeated for every 100bp interval up to 5000 for each comparison
